# Boosting underwater image quality: a deep learning approach to denoising and enhancement

**DOI:** 10.1038/s41598-026-47888-7

**Published:** 2026-04-14

**Authors:** Najaf Ali, Muhammad Habib, Fahad Burhan Ahmad, Saif Ur Rehman, Yuelin Guo, Ahmad Alshammari

**Affiliations:** 1 University Institute of Information Technology, PMAS Arid Agriculture University, Rawalpindi, Pakistan; 2 Institute of Cyberspace Security, Harbin Institute of Technology, Shenzhen, Shenzhen, 518055 China; 3 Department of Computer Sciences, Faculty of Computing and Information Technology, Northern Border University, Rafha, 91911 Kingdom of Saudi Arabia

**Keywords:** Underwater image restoration, Deep learning, DnCNN, Denoising, Image enhancement, Attention mechanism, Multi-color space, EUVP, LSUI, Real-time processing, Engineering, Mathematics and computing, Ocean sciences

## Abstract

Underwater image restoration is significant for various applications such as ecological evaluation, exploration, searching and rescue operations, and autonomous vehicle navigation. In underwater environments, spatial images are frequently degraded as a result of light scattering, absorption, sensor noise, and reduced contrast. This research proposes a whole framework with deep learning that simultaneously performs restoration and enhancement. At a single stage, solving the problems of underwater image degradation in a holistic approach. The core of the proposed approach in this study is a Denoising Convolutional Neural Network (DnCNN) architecture. Where the suppression of noise takes place with extreme focus on significant detail by an advanced non-local attention mechanism. For the further natural color restoration, multi-color space transformations RGB, LAB, and HSV. Which come into play for enhancing the contrast adjustment and color correction of contrast and a correction of colors enabling effective correction of deep-sea views. The framework takes advantage of both synthetically modified images and actual underwater images for model training which offers enhanced generalization for various settings. For the evaluation of the developed approach, two datasets of underwater images, EUVP and LSUI, were used. For the evaluation of the developed approach, two datasets of underwater images, EUVP and LSUI, were used. For the EUVP dataset, this model produces a PSNR of 30.77 dB, an SSIM of 0.892, RMSE of 0.065, and NIQE of 3.52. It produces a PSNR of 29.90 dB, SSIM of 0.881, RMSE of 0.071, and NIQE of 3.82 for the LSUI dataset. As mentioned earlier these results outperform the baseline DnCNN model and show consistent performance with varying underwater conditions. Accompanying the performance results, the model achieves high fidelity results while being lightweight, real-time processing.

## Introduction

**Fig. 1:**
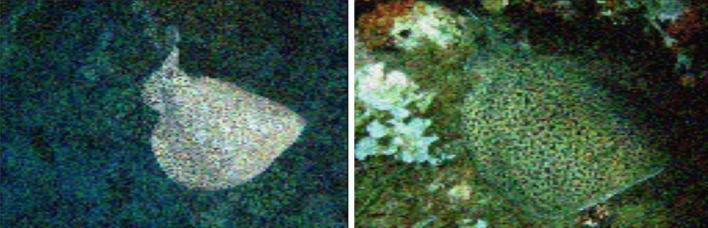
Underwater noisy image.

Underwater image denoising and enhancement lies at the intersection of computer vision and deep learning, which would enhance the level of image quality obtained in underwater environments. These photos tend to exhibit in Fig. 1 degradations including noise, limited contrast, haziness, and extreme color distortions since the light is absorbed and scattered by the water^1–4^. Denoising refers to the process of reducing noise caused by sensors, by suspended matter particles, or ambient conditions whereas enhancing entails improving clarity, sharpness and red-green balance to achieve a more advantageous visualization^5–7^. Convolutional Neural Networks (CNNs) and Generative Adversarial Networks (GANs) deep learning models were used, which are popular to solve these problems. Developments in the recent past keep extending the frontiers of the enhancement of underwater imagery. As an example, WaterCycleDiffusion enhances the quality of perceptual images because of visual-textual fusion by diffusion models^8^, and histogram similarity-based methods would offer strong color compensation by multi-attributes adjustment^9^. Cross-domain methods are also valuable to note: S2G-GCN can be used to provide spectrum-to-graph classification^10^, reinforcement learning can be used to jointly detect and track objects in radar systems^11^, as well as multilayer fusion with self-organized stitching can be used to improve the reconstruction of scenery clearing^12^.

Similarly, visual-textual mutual guidance fusion networks have shown promise in remote sensing visual question answering^13^, demonstrating the broader applicability of multimodal fusion architectures that combine different data representations a concept that parallels our multi-color space attention fusion approach. Such a variety of strategies in a nutshell makes the relevance of hybrid architectures—domain knowledge and adaptive learning—practices which make sense in our context visibility by integrating physics-inspired color correction with deep attention systems. CNNs are superior at hierarchical feature extractions to remove distortions whereas GANs are best at restoring fine textures and colors through adversarial learning. Some of them improve results by performing transformations among a small number of colors spaces^3,14^, whereas others use attention mechanisms to emphasize salient points and deemphasize background clutter^15–18^. Combination schemes involving a denoising and enhancing algorithms have recently been developed and produce realistic looking underwater pictures that can be used in applications such as marine exploration, autonomous navigation and monitoring of aquatic ecosystem^2,4,19^. The image restoration algorithms in the underwater image restoration category could be broadly divided into the categories of model-based and categories. Model-based approaches are based on light propagation physics, and one of the commonly used models is the JaffeMcGlamery model^2,19,20^. This model mathematically presents light attenuation and scattering in water as represented in the following Eq. (1).

1 $$I\left(x\right)= J\left(x\right)\cdot t\left(x\right)+ B\left(x\right)\cdot \left(1 - t\left(x\right)\right)$$

with I(x) being the observed degraded image, J(x) being the radiance of the scene, t(x) being the transmission map that will be used on the modeling of attenuation of light, and B(x) being the background scattering. Although they are physically explainable, such techniques assume historical background of underwater situations and hence they are generalized. The intensity redistribution is instead the focus of histogram-based techniques. As an illustration, Adaptive Histogram Equalization (AHE) and its variant CLAHE produce brighter and higher contrast when the environment is turbid^1,6,7^. Filtering based algorithms use either median, bilateral, or wavelet based filters to reduce noise without edge degradation^1,4,20^. The enhancement based on retinex enhances visibility by simulating human vision, fine-tuning local contrast and brightness in order to remove details^1,6^. But these methods of the past when put under varied underwater environments fail more often because they are not naturally adaptable.

The learning-based approach transition has made key strides to improve the restoration of underwater images. CNNs, GANs, and Vision Transformer (ViT) are techniques in deep learning that are capable of automatically learning noisy to clean image mappings on large datasets [5, 8–9, 13 R1-R2]. CNN-based models take advantage of convolutional kernels in order to learn both local and global features whereas GAN-based models such as Sea-Pix-GAN rebuild realistic textures based on adversarial refining outputs^7,21,22^. Multi-color space algorithms conversions to the space including HSV and LAB to enhance correction of attenuation of colors^3,14^. Fusion-based techniques enhance images using multiple strategies and then merge them with weighted optimization^5,21,23^. Attention mechanisms further improve restoration by allocating more focus to essential features while suppressing background noise^15,17,18,24^. Recently, diffusion-based models such as DiffWater employ iterative conditional denoising to refine underwater images step by step, achieving state-of-the-art results^25^. Hybrid frameworks, such as combining DnCNN or PDAN for denoising with MCRNet or Sea-Pix-GAN for enhancement, have shown superior performance by addressing multiple distortions simultaneously^7,21,22^.

From a mathematical perspective, denoising and enhancement can be formulated as learning a function ${F}_{\theta }$, parameterized by deep networks, that maps degraded underwater images ${I}_{d}$ to restored outputs ${I}_{r}$ as shown in Eq. (2).

2 $${I}_{r}={F}_{\theta } ({I}_{d})$$

*where θ* represents the learnable parameters optimized to minimize a loss function. Common image processing techniques such as histogram equalization^6,7^, anisotropic diffusion filters^1,6,19^ and wavelet-based denoising^20^ offer informative details but have no strong standings in the actual underwater setting. In comparison, deep learning-based models are superior to them because they take advantage of massive data sets and automatically extract domain-specific features^5,16,17,26^. In particular, the CNN-based architectures such as DnCNN learn the distortion of noise type and thus perform better at denoising^6,7,16,17^. GAN-based models are examples like Sea-Pix-GAN, which end up producing visually improved results through learning complex mappings between noisy and clean distributions^7,21,22^. The images under water are compressed and restored by autoencoders to remove the noises^5,7,17^, whereas Vision Transformers (ViTs) handle the global aspects to improve the smoothness of colors and structure^15,18,26^. Attention based techniques enhance visual clarity because they best optimize the fine-tuning of prominent features^16–18^. The diffusion models, such as DiffWater, refine images step by step by performing the refinements step by step^25^. The approaches reflect that deep learning exceeds the traditional algorithms in adjustability and generalization by a wide margin especially in cases of high level of variation in the underwater environments^1,5,16,19^.

In spite of these benefits, deep learning-based approaches are notable because they have major challenges. They require vast quantities of annotated data, which is limited to underwater conditions^5,16,21,26^. When training on a small or biased set of data, overfitting can occur as well as subpar generalization^5,16,17^. Computational needs are also demanding and thus it is difficult to deploy it in real time systems^5,17,26^. Besides, deep models are frequently difficult to understand in contrast to physics-based methods^6,20^. Further impairment of performance is caused by extreme underwater conditions including high turbidity or swift illumination changes. However, the possible solutions to them, including PDAN and Sea-Pix-GAN, using transfer learning and domain adaptation, should enhance their adaptability and efficiency^5,7,16,21,22,26^.

The suggested framework will overcome these restrictions by applying a strong and flexible deep learning approach to underwater image restoration. It uses denoising generators like DnCNN or PDAN and enhancement generators like MCRNet or Sea-Pix-GAN to both reduce noise and output perceptual quality^7,21,22^. The framework can maintain structural details and increase the accuracy of colors by using residual learning, multi-color space transformations, and attention mechanisms^3,14,15,18^. Optimization of the models by using the hybrid loss functions that determine the correctness of the pixels at the pixel level and the perceptual truth with maximum effectiveness is called model optimization. This method has not only the advantage of aesthetically appealing results but also allows real-time execution because of support through systems like Google Colab that allows not only the ability to deploy the results in mass deployment but also to use it in the underwater applications in research and in reality as well^5,17,26^.

The study paper highlights the development of a framework of SSI image restoration based on a deep learning architecture with the DnCNN architecture and other image quality improvement techniques. The produced model involves introducing the application of residual learning and multi-color space transformation in the framework of attention-based models to restore underwater images with high structural accuracy. The most important contributions of the research works toward the paper have been stated below:

1. Residual Multi-Color Attention Fusion: A new method that combines RGB, HSV and LAB features adaptively to generate better structural and color reconstruction with the help of maximum attention maps that are learned.
2. DWT-IDWT Residual Feature Learning: DWT based features are integrated into residual blocks and used to extract hierarchical features at the highest frequency detail and the lowest noise is estimated.
3. Hybrid Noise-Perceptual Loss Function: Losses The hybrid pixel-level and perceptual loss is based on multi-color features, with the aim of improving visual quality without affecting structural fidelity.
4. Lightweight Attention-based Fusion: Real-time Adaptability Algorithm: This can be used to deploy attention-based fusion in practice without significant visualization loss.

## Literature review

The recent research of underwater image denoising and enhancement has been motivated by a rather bigger source of adding preprocessing, augmentation and deep learning models in a single model in order to increase the robustness against harsh aquatic conditions. In addition to traditional methods, other current studies feature multimodal fusion^8^, histogram-based compensation^9^, graph convolutional networks^10^, reinforcement learning^11^, and multilayer stitching^12^, demonstrating the fact that the field is currently developing more refined, task-familiarized methods of improvement^27^ presented a combined approach wherein the preprocessed steps of edge enhancement, brightness correction, and color correction were followed by CLAHE-based contrast correction. The images were inputs into a DnCNN model for noise removal, whereas the SURF features were extracted for facilitating object detection. Testing over several underwater datasets by means of PSNR, SSIM, and MSE verified the finding wherein the combined approach operated better than singular ones with a result of detecting 62.02 for DnCNN as opposed to 52.02 for CLAHE, thereby highlighting the potential of combined approaches for the underwater mission.

A persistent issue when creating learning-based underwater improvement schemes is the unavailability of consistent ground-truth databases. In^28^ Saini et al., described a reference set of 3,000 images of the shallows taken at depths less than five meters, which were used as training media for the UMLDnet. This CNN-based architecture combined minimum-loss dehazing using scene radiance and transmission maps, reducing scattering and absorption distortions UMLDnet showed to be more effective to real-world applications of resources: it converged quickly, had a reduced computational cost, and better PSNR compared to traditional in the field of deep learning.

As the scattering causes intense degradation of optical underwater images, sonar images have other challenges of noise like speckle distortion, which affect detection accuracy in robotics. In^29^ Wang et al. compared nine deep denoising models on five sonar datasets, and learned to adapt to sonar data optical denoising methods. They then suggested a multi-frame denoising approach, in which the result of one model was used to complement another model. This ensemble technique was used to dramatically enhance sonar object recognition which indicates that it is a model that can be transferred to other imaging modalities.

Healthy works on the improvement of the underwater optical images have also been centered on better architectures. In^30^ the Wang et al., presented OUNet-JL which is an optimized U-Net system that added a Multi-Residual Module (MRM) to enhance the ability of the system to represent detail and a Strengthen-Output-Subtract Feature Reconstruction Module (SOSFM) to enhance the signal-to-noise ratio and minimize blur. Channel attention mechanism helped remove color imbalance in channels mainly red and blue. Using UIEB and UFO-120 training set, OUNet once again outperformed baseline models in terms of PSNR, SSIM, and perceptual visual quality, and the advantages of adaptive residual and attention mechanisms.

Nonetheless, there are other methods that have attempted to combine spatial and deep learning. In^31^, Priyadharshini et al., came up with an ensemble framework that combines spatial enhancement and CNN-based learning to counter over-saturation and preserve texture. Tried on UIEB and EUVP datasets, the framework demonstrated relatively high improvements in but less robust generalization during unseen characteristics, which indicates the shortcomings of ensemble procedures in the absence of specific adaptation modules.

One of the environments has been the adoption of prior knowledge to network architectures. The Deep Unfolding Network (DUN) introduced in^32^ Lei et al., combined physics into the deep features learning. The attenuation priors’ wavelength-dependency were added to its Color Prior Guidance Block (CPGB) and the Nonlinear Activation Gradient Descent Module (NAGDM) and Inter-Stage Feature Transformer (ISF-Former) enhanced the optimization stability. This hybrid design department at least has state-of-the-art performance on a few data sets, beating CNN and GAN baselines in PSNR and perceptual metrics, and being interpretable due to its physics-based nature.

The comprehensive surveys that have been conducted have been instrumental in cementing progress in such areas. In^33^ Zangana et al., authors have completed a review of deep learning-based image improvement in underwater, medical, and remote sensing and concentrated on CNNs, GANs, and Autoencoders. They also laid stress on computational issues, large labeled data needs, and performance criteria—PSNR, SSIM, NIQE, and UIQM. This high-level appraisal put into focus the variability of the deep models and emphasized the necessity of lightweight architecture technology and unsupervised training methods.

Focusing on condition-based degradations^34^, came up with a model where specialized networks were trained to those types of degradation namely low illumination, haze and color imbalance. Accordingly, subsets of UIEB and EUVP datasets were torn out so that each network would narrow its focus to mitigating one major factor. This condition-adaptive training was far much more successful than generic models especially in SSIM and NIQE scores which is a testament to the fact that specialization, when performed purposefully, can result in increased fidelity underwater reconstructions.

Ref^35^ have proposed UWIE-Net, a parallel CNN architecture that is a denoising method that is based on blind convolution and wavelet fusion. CNN module was used to improve the contrast and textures whereas blind convolution removed non-signal. Wavelet decomposition was used to fuse outputs in order to maintain edges. On the UIEB dataset, UWIE-Net achieved an MSE of 23.5, PSNR of 34.42, AG of 13.56, PCQI of 1.23, and UCIQE of 0.83 which was better than the competing models and provided sharper images that were better balanced in color.

The UW-DiffPhys framework presented by^36^ was a step forward in the domain since it combined denoising diffusion probabilistic models (DDPMs) with physics-based enhancement that commonly requires minimal training. The model used a denoising U-Net in addition to Denoising Diffusion Implicit Models (DDIMs) so as to reduce the time of inference. This architecture maintained the diffusion model stability and minimized the computing load. Quantitative comparisons with the traditional schemes showed that merging techniques of generative diffusion with physical priors yield higher PSNR, SSIM, and UIQM, and both these methods are accurate and efficient in terms of real-time performance on underwater imaging.

The literature review also presents the ongoing advancements in underwater image denoising and enhancement with the preprocessing technique that has continued to develop to deep learning models that incorporate priors, residual modules, and generative techniques. Although Table 1 shows substantial advancement in PSNR, SSIM, and visual fidelity using state-of-the-art structures, issues of computational complexity, inability to generalize to the environment and adapt to real-time conditions in underwater environments have not been addressed. To overcome these shortcomings, the current study has established a DnCNN-driven model, which combines residual learning with transformations of multi-color spaces (RGB, LAB, HSV), and attention mechanism to ensure effective restoration. Moreover, the suggested design focuses on the applicability in real time via Google Colab, which will guarantee the efficiency of computation, as well as its accessibility. With the combination of the above advances, the study can contribute to the existing literature but provide a viable and efficient method of enhancement of underwater image.

**Table 1:** Summary of existing state of art methods.

Reference	Dataset	Methodology	Performance	Limitations
UIR-PolyKernel^37^	Benchmark datasets	Polymorphic Large Kernel CNNs, Hybrid Domain Attention	State-of-the-art performance	Parameter-heavy models, Limited to benchmark datasets
GuidedHybSensUIR^38^	Real-world underwater datasets	Color Balance Prior Guided Hybrid Sense Framework, Detail Restorer, Feature Contextualizer	Outperforms 37 methods overall	Not best in certain individual cases, Complexity in multi-scale sensing
DUN^32^	Multiple underwater image datasets	Deep Unfolding Network (DUN), Color Prior Guidance Block, Nonlinear Activation Gradient Descent	Superiority over state-of-the-art methods	Lack of explicit physical process incorporation, Computational complexity
FUGN ^39^	Extensive experimental validations	Fusion-based Underwater Graph Network (FUGN), Sobel and Gaussian Blur operators	Improvements in FSIM, PSNR, SSIM	Dependency on initial block segmentation, Complex fusion of CNN and GNN architectures
UW-Net^40^	Well-known datasets	UW-Net with DWT and IDWT, Chromatic Adaptation Transform Layer	Enhanced performance across metrics	Limited to DWT-based feature extraction, Potential color fidelity issues in practical applications

## Proposed methodology for underwater image denoising and enhancement

**Fig. 2:**
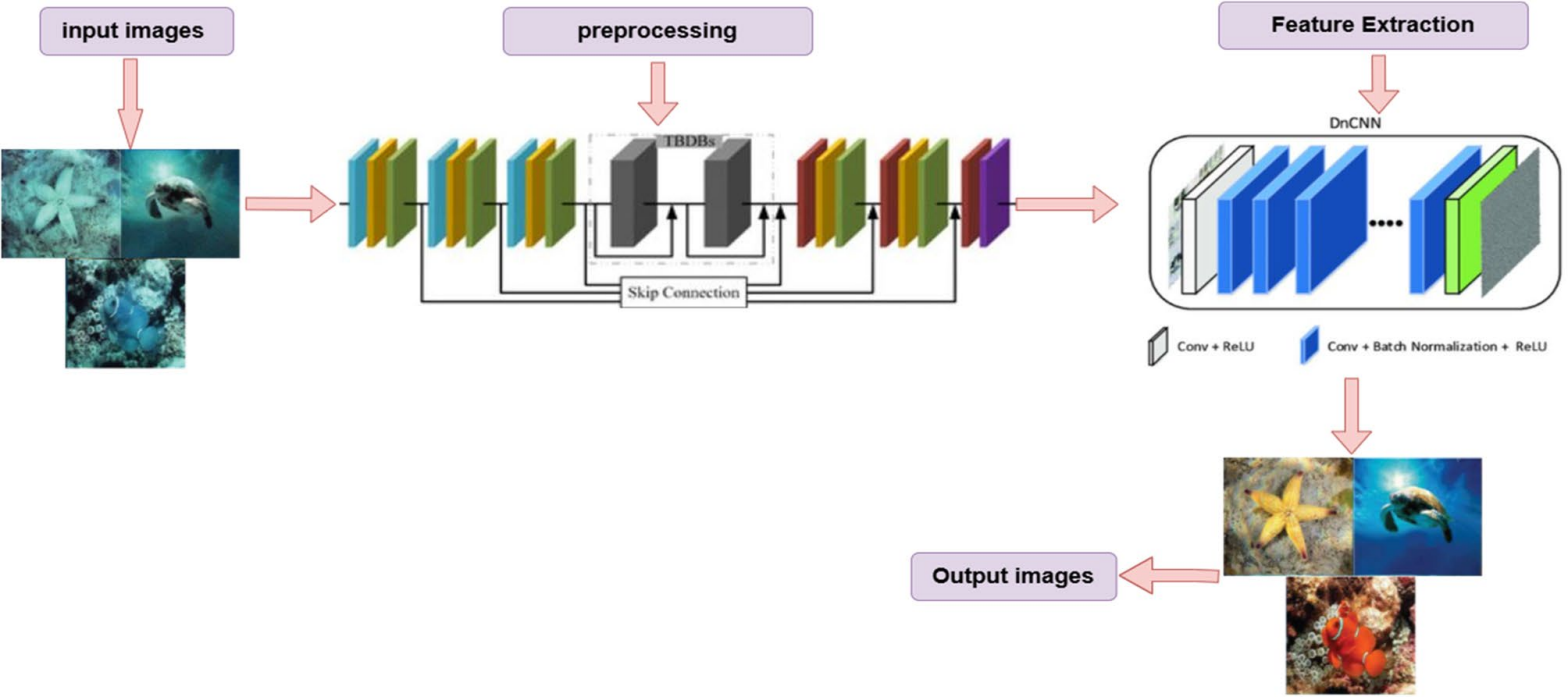
Underwater Image Denoising and Enhancement proposed model using DnCNN.

The proposed methodology will combine a deep learning architecture, which is referred to as DnCNN with residual learning to restore underwater images. The transformations of multi-color space (RGB, LAB, HSV) are used to improve color faithfulness and contrast. Also, the attention mechanisms are included to highlight attention areas and silence the background noise to enhance structural precision. Many problems like noise and low contrast also compromise quality in the situation of images taken in the underwater conditions. The colors are also distorted due to light scattering and light absorption. Underwater visibility and clarity are important not only in the exploration of the sea, but in the study of robotics and the ocean. All these activities are associated with difficulties that are caused by low visibility. Thus, the central goal of our study is to enhance the quality of these underwater images, with the help of the deep learning techniques, especially, the DnCNN image denoising convolutional neural network. This approach that has been described in detail is based on model architecture design, dataset construction, addition of noise, evaluation, and training. All the experiments were run 5 times each individually and the results are expressed as mean +—standard deviation to achieve statistical reliability. It uses new images which have rich convolutional levels wherein noise is eliminated by residual learning without losing key edges and details of the image. This method also fulfills the demands of the image structural consistency and naturalness. The model can also perform reliably in the real underwater environment with the help of controlled training and testing. The approach uses the simulation of the synthetic environment by augmenting noise with the objective of providing effectiveness in a wide range of underwater conditions. In this section, the detailed workflow of the entire data collection process up to and including the implementation of the models is explained with all the phases of execution being highlighted in Fig. 2.

### Unified optimization framework

#### Integrated optimization framework

In order to streamline the proposed underwater image restoration model, we formulate a single loss function that compliantly involves denoising, color correction and chromatic enhancement. To practically eliminate the undesired distortions created by underwater the total loss L total is the sum of three losses: the DnCNN denoising loss LDnCNN, the color correction loss LCC, and the chromatic layer loss LCL Color correction loss is defined as the L 1 distance between the color-corrected result and the clean reference, the color fidelity is improved. The chromatic layer loss uses structural similarity (SSIM) to preserve perceptual and structural details. The cornerstone loss identity can be written as Eq. 3:

3 $$L_{total} = \, \lambda_{{_{1} }} L_{DnCNN} + \lambda_{2} L_{CC} + \lambda_{3} L_{CL}$$

where l1, l2 and l3 are the weighting coefficients used to look after the contribution of every component. The formulation permits concomitant image de-noising, color recovery and structural empowerment thereby guaranteeing hearty improvement of underwater image, comprised of a wide range of datasets.

### Dataset collection and preparation

A wide and rich dataset is needed in order to perform successful underwater image denoising and enhancement. In Table 2 this research, EUVP and LSUI are utilized. EUVP offers both noisy-clean and unpaired underwater image, manipulated under different depth, under different light and turbidity conditions and similar and different environments, thus supervised and unsupervised learning can be conducted in UVP. LSUI has annotated and non-annotated pictures on different aquatic spaces, and problems such as color deformity, decrease in contrast, scattering, light uptake, and aid in model generalization in unseen conditions. Manual methods are used to upload all the images into a systematized folder (dataset/). The photographs are used to provide an image in a standard size of 256 × 256 pixels, where the BGR images are converted into the RGB images using the OpenCV to guarantee the appropriate color data. All values in the range [0, 1] are scaled such that numerical instability that might be encountered during training is reduced (pixel normalization) and is given by should be expressed as in Eq. (4).

4 $${I}_{norm}= \frac{I- {I}_{min}}{{I}_{max}- {I}_{min}}$$

*where* $I$ is the image intensity and ${I}_{max}$, ${I}_{min}$ are the maximum pixel values and minimum pixel values respectively. To enhance the vulnerability of the models, clean images are also added with synthetic noise to replicate the distortions of the underwater like Gaussian noise. This training is done to give adequate variation in terms of color, noise, and scene conditions, which was a reliable basis of training deep learning models that would be capable of decomposing and enhancing under-water image quality to a high standard.

**Table 2:** Dataset description.

Dataset Name	Dataset type and description	Web-link
EUVP	• Provides both paired (noisy-clean) and unpaired underwater images • Covers diverse scenes with varying depth and lighting conditions	https://github.com/xinzhichao/Underwater_Datasets?tab=readme-ov-file
LSUI	• Offers unpaired real-world underwater images with noise and distortion • Simulates challenging environments for robust model testing	https://www.kaggle.com/datasets/noureldin199/lsui-large-scale-underwater-image-dataset

#### Noise simulation for realistic underwater conditions

To better approximate real underwater degradation caused by light scattering, absorption, and turbidity, we simulate multiple types of noise beyond the basic Gaussian model. The degraded image ${\mathrm{I}}_{\mathrm{noisy}}$ is generated by contaminating clean images ${\mathrm{I}}_{\mathrm{clean}}$ with the following noise models:

**Gaussian Noise:** Models sensor noise and thermal fluctuations in Eq. 5:

5 $${I}_{noisy}={I}_{clean}+{\eta }_{g},{\eta }_{g}\sim N(0,{\sigma }^{2})$$

where the standard deviation σ is randomly sampled from [15/255, 35/255] during training.

**Speckle Noise**: Simulates coherent imaging artifacts common in underwater sonar and laser systems in Eq. 6:

6 $${I}_{noisy}={I}_{clean}+{I}_{clean}\times {\eta }_{s},{\eta }_{s}\sim N(0,{\sigma }_{s}^{2})$$

where ${\upsigma }_{s}$ is set between 0.1 and 0.3 based on typical underwater characteristics.

**Salt-and-Pepper Noise**: Represents impulse noise from transmission errors. The noisy image is generated in Eq. 7:

7 $${I}_{noisy}(x,y) = \left(1 - M\left(x,y\right)\right).{I}_{clean}(x,y) + M\left(x,y\right). S(x,y)$$

where M(x, y) ∈ {0,1} is a random binary mask, where 1 indicates the pixel is corrupted and 0 means the pixel remains clean. S(x, y) ∈ {0,255} randomly assigns salt (255) or pepper (0) to corrupted pixels. The noise density p (probability of M(x, y) = 1is randomly selected from [0.01, 0.05]. During training, one or a combination of these noise types is randomly applied with probability 0.5 to augment the dataset. This ensures that the model is exposed to diverse underwater degradation patterns, improving its robustness and generalization capability in real-world conditions.

### Preprocessing and data augmentation

Each of the input images is first translated into the RGB format so that all input images in the dataset would share the same color representation. The size of each image is then downsized to 256 × 256 pixels to ensure that the sizes of the images match in batch training. Once the pixels are resized, pixel normalization is done by the division of the pixel values with 255, which directly proportionally normalizes the pixel intensity range [0, 1]. Synthetic Gaussian noise is used to make the clean images appear noisy like underwater noise. The mean value and standard deviation of the noise is also s = 25/255. This process can be mathematically defined as in the Eq. (8).

8 $${I}_{noisy}= {I}_{clean}+\eta , \eta \sim \mathcal{N}(0, {\sigma }^{2})$$

The input clean image ${\mathrm{I}}_{\mathrm{clean}}$, and the output noisy image ${\mathrm{I}}_{\mathrm{noisy}}$ distinguish the noise s e where e is a sample of noise of a normal distribution with mean value e and variance value ${\upsigma }^{2}$. They are also data augmented, flipped, rotated, and adjusted in brightness, to make the datasets more diverse, and decrease overfitting. This preprocessing and augmentation is necessary to inform the DnCNN model of different realistic underwater distortions and make it more capable of mapping to images it has never seen and conducting appropriate image denoising and image enhancement.

#### Simulating realistic underwater distortions

Although Gaussian noise provides a baseline for simulating general underwater degradation (as detailed in Sect. “Noise Simulation for Realistic Underwater Conditions”), the actual underwater environment exhibits a broader spectrum of noise characteristics. As described in Sect. “Noise Simulation for Realistic Underwater Conditions”**,** we simulate three distinct noise types during data augmentation: Gaussian noise (σ ∈ [15/255, 35/255]), speckle noise (${\upsigma }_{\mathrm{s}}$ ∈ [0.1, 0.3]), and salt-and-pepper noise (p ∈ [0.01, 0.05]). These models capture sensor noise, coherent imaging artifacts, and impulse distortions respectively.

In addition to these noise models, we apply synthetic color decay based on simulated light absorption curves and local blurring to replicate scattering effects. The pixel intensities of RGB channels are adjusted using wavelength-dependent attenuation coefficients. This comprehensive augmentation strategy guarantees that the model encounters a wider variety of realistic underwater distortions, enhancing generalization and resilience in real-world applications.

#### Physically realistic underwater degradation

To approximate the natural underwater environment, the noise models in Sect. “Noise Simulation for Realistic Underwater Conditions” are complemented by a physics-based image formation model accounting for wavelength-dependent absorption, scattering, and turbidity. The degraded image is represented as in Eq. 9:

9 $$\mathrm{I}(\mathrm{x}) =\text{ J}(\mathrm{x})\cdot \mathrm{t}(\mathrm{x}) +\text{ B}\cdot (1 -\text{ t}(\mathrm{x}))$$

where $I(x)$ is scene radiance, B is background light, and $t(x)$= $B\cdot (1 - t(x))$ is the medium transmission for each color channel (R, G, B). To create a comprehensive degradation simulation, we cascade sensor-based noise (Gaussian, speckle, and salt-and-pepper from Sect. “Noise Simulation for Realistic Underwater Conditions”) with physical underwater effects in Eq. 10:

10 $${\mathrm{I}}_{\mathrm{final}} =\mathrm{P}(\mathrm{N})({\mathrm{I}}_{\mathrm{clean}}$$

where N represents noise types from Sect. “Noise Simulation for Realistic Underwater Conditions” and P represents the physics-based attenuation model. This cascaded approach ensures training on images exhibiting both statistical noise characteristics and physical distortions. The model is trained and tested on real paired underwater data (EUVP and LSUI) alongside synthetic degradations, with quantitative metrics (PSNR, SSIM, RMSE) and visual comparisons validating performance under physically realistic conditions.

### Color correction (multi-color space + attention)

Underwater images also suffer from the color distortion caused by absorbing and scattering lights, which acts unevenly upon the R, G, and B channels. To correct this, we apply multi-color space transformations (RGB, HSV, LAB) combined with an attention mechanism to adaptively enhance each channel’s contribution. Assume a degraded input image ${I}_{c\left(x,y\right)}$, c ∈ {R, G, B} the original RGB color correction is defined as in Eq. (11).

11 $${I}_{c\left(x,y\right)}{\prime}= {I}_{c\left(x,y\right)}+ {\lambda }_{c}\cdot {\theta }_{c}\cdot {A}_{c }\left(x,y\right)$$

*where* ${\lambda }_{c}$ = channel-specific scaling constants, ${\theta }_{c}$ = scaling factor determined from channel means, ${A}_{c }\left(x,y\right)$ = attention map highlighting critical color features for adaptive enhancement.

The Gray World Hypothesis also assumes the average color of the natural scene as gray, i.e., the red, green, and the blue channel means would all ideally be equal. To correct color distortions in underwater images, scaling factors for the red and the blue channels are calculated relative to the green channel. The correction factors are defined in Eq. 12:

12 $${\theta }_{r}= {(\overline{I } }_{G}-{\overline{I } }_{R})\cdot (1-\frac{{I}_{R\left(x,y\right)}}{{I}_{G\left(x,y\right)}}) , {\theta }_{b}= {(\overline{I } }_{G}-{\overline{I } }_{B})\cdot (1-\frac{{I}_{B\left(x,y\right)}}{{I}_{G\left(x,y\right)}})$$

*where* ${\overline{I } }_{R}$​ , ${\overline{I } }_{G} , {\overline{I } }_{B}$ are the average Intensities of red, green, and blue channel respectively, while ${I}_{R\left(x,y\right)}$ , ${I}_{G\left(x,y\right)} , {I}_{B\left(x,y\right)}$ and denote the pixel intensities at spatial position? By applying the scaling factors ${\theta }_{r}$ ​ and ${\theta }_{b}$ from Eq. (12), the red and blue channels are corrected relative to the green channel. This adjustment compensates for underwater attenuation, thus improving the color balance and restoring a more natural appearance to underwater images.

#### Multi-color space transformation

In pursuit of enhanced color fidelity, perceptual brightness when recording an image in underwater, the proposed method utilizes transformations in several color spaces, combines attention and ultimately blends the outputs to recreate the image. Here HSV In HSV the initial RGB image is converted to the HSV image color space to be highlighted by the hue and saturation which in underwater image circumstances tends to be distorted and displayed in Eq. (13).

13 $${I}_{\left\{HSV\right\}}\left(x,y\right)= RGB2HSV\left({I}_{\left\{RGB\right\}}{\prime}\left(x,y\right)\right)$$

*Here*, ${I}_{\left\{RGB\right\}}{\prime}\left(x,y\right)$ denotes the preprocessed image of the RGB image, and corresponding image in HSV space. This transformation allows better manipulation of hue and saturation components to restore natural-looking colors.

RGB image gets converted into the LAB color space LAB color space to enhance perceptual quality. This transformation focuses on improving luminance (brightness) and overall visual contrast as shown in Eq. (14).

14 $${I}_{\left\{LAB\right\}}\left(x,y\right)= RGB2LAB\left({I}_{\left\{RGB\right\}}{\prime}\left(x,y\right)\right)$$

*Here*, ${I}_{\left\{RGB\right\}}{\prime}\left(x,y\right)$ signifies the preprocessed RGB image, while ${I}_{\left\{LAB\right\}}\left(x,y\right)$ denotes the corresponding LAB image. The L-channel contains luminance (brightness), and color-opponent dimensions are captured by the A and B-channels. This transformation enhances luminance perception and allows for improved contrast correction in underwater images. To emphasize significant structure and chromatic attributes, attention is concentrated in the HSV and LAB domains. It provides a higher weight for informative regions and shrinks the irrelevant ones as shown by the Eq. (15).

15 $${I}_{\left\{HSV\right\}}{\prime}= {I}_{\left\{HSV\right\}}\odot {A}_{\left\{HSV\right\}}, {I}_{\left\{LAB\right\}}{\prime}= {I}_{\left\{LAB\right\}}\odot {A}_{\left\{LAB\right\}}$$

In which the element-wise multiplication is denoted by ⊙. In this case, ${A}_{\left\{HSV\right\}}$ is the matrix of channel attention that have been trained in the preparatory phase, and ${I}_{\left\{HSV\right\}}{\prime}$ and ${I}_{\left\{LAB\right\} }{\prime}$ are modified outputs. These attention mechanisms allow the network to concentrate on salient visual features, including edges and other structural details and inhibit less informative areas. Lastly, the augmented RGB snapshot is rebuilt by the integration of features across several color spaces. These combinations are incorporated in the fusion process to come up with a natural balanced output based on RGB, HSV, and LAB features as illustrated in Eq. (16):

16 $${I}_{\left\{final\right\}}{\prime}= \alpha {I}_{\left\{RGB\right\}}{\prime}+ \beta \cdot HSV2RGB\left({I}_{\left\{HSV\right\}}{\prime}\right)+ \gamma .LAB2RGB\left({I}_{\left\{LAB\right\}}{\prime}\right)$$

The fusion weights α, β and γ are learnable parameters optimized via backpropagation using a softmax function over three learnable scalars: α, β, γ = softmax Initialized to [1/3, 1/3, 1/3] by setting α = β = γ = 0, they are updated alongside network parameters using Adam. After training on EUVP, the weights converge to γ = 0.31, β = 0.35, γ = 0.34, indicating adaptive prioritization of HSV and LAB spaces for color correction while preserving RGB structure. This learnable mechanism ensures balanced fusion for uniform color, structural integrity, and perceptual improvement across varied datasets. The algorithm 1 explains the preprocessing and color correction stage that was used when improving the quality of underwater images. The pictures are re-scaled to a regular resolution, and finally changed to RGB. Gaussian noise is employed to clean samples to replicate distortions which affect underwater images before augmentation. The subsequent operations include flipping, rotation and change of brightness to make the dataset diverse. The pixel normalization is employed to have consistent training where intensity values are kept in a stable range. The algorithm converts the images into LAB color space, HSV color space and RGB color space so as to extract the complementary features. The attention mechanisms and Gray-World correction are utilized in order to remove the color imbalance and underline the necessary details. The last step of algorithm 1 is the integration of the corrected outputs of all spaces, which form better inputs to use the denoising process.

##### Algorithm 1:

Underwater image preprocessing and color correction.


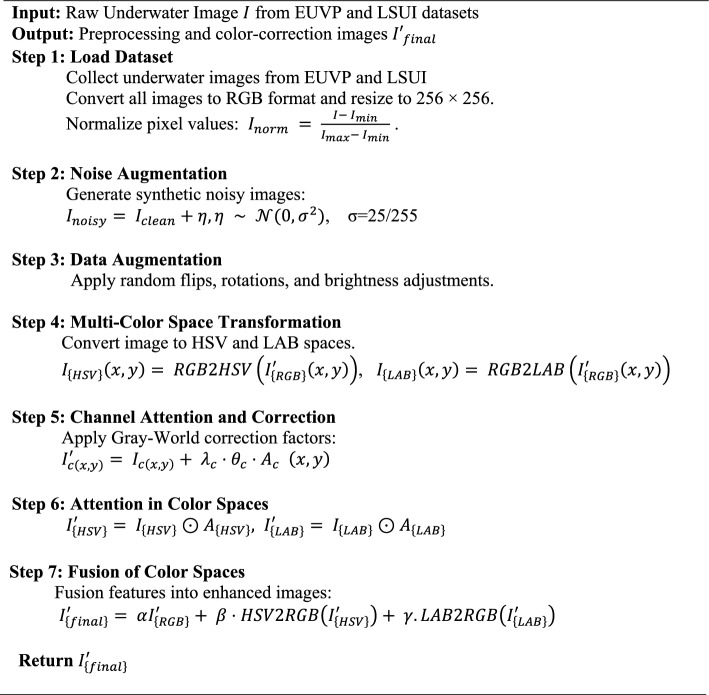


### Non-local attention mechanism

The presented model adopts a non-local attention mechanism in order to overcome the local dependencies that are implemented on the whole feature map. The network does not just use local convolutions as it would be but instead calculates a similarity score between each position of the feature map and places more emphasis on those regions that are important in the restoration of the image. This enables the model to accentuate the edges, textures and salient areas and filter off the background noise that is not important thus enhancing preservation of structures and color support of underwater images. Learning of the attention maps is an adaptive process and the effect of the attention maps is evident in the superior results in denoising and enhancement.

Mathematically, the non-local attention operation is defined in Eq. 17:

17 $${\mathrm{y}}_{\mathrm{i}} = \frac{1}{\mathrm{c}}\sum_{\mathrm{j}}\mathrm{f}({\mathrm{x}}_{\mathrm{i}}, {\mathrm{x}}_{\mathrm{j}})\text{ g}({\mathrm{x}}_{\mathrm{j}})$$

where i is the output position, j represents all positions, and x is the input feature map. The function $g({x}_{j})$= W ${x}_{j}$ is a simple linear transformation, and f $({x}_{i}, {x}_{j})$ measures similarity between ${x}_{i}$ and ${x}_{j}$.We adopt the embedded Gaussian formulation in Eq. 18:

18 $$\begin{gathered} f\left( {X_{i} ,X_{j} } \right) = e^{\theta } (X_{i} )^{t} \phi X_{j} \hfill \\ \theta X_{i} = w\theta X_{i} \hfill \\ \phi X_{j} = w\theta X_{j} \hfill \\ \end{gathered}$$

and the normalization factor is defined in Eq. 19:

19 $$\mathrm{cx}= \sum_{\mathrm{j}}\mathrm{f}({\mathrm{x}}_{\mathrm{i}}, {\mathrm{x}}_{\mathrm{j}})$$

Finally, the non-local block is incorporated using a residual connection in Eq. 20:

20 $${\mathrm{z}}_{\mathrm{i}} ={\mathrm{w}}_{\mathrm{z}}{\mathrm{y}}_{\mathrm{i}} +{\mathrm{x}}_{\mathrm{i}}$$

### Wavelet transform

Wavelet analysis it becomes a resource for image processing, enabling the breakup of an image into two key components: the approximations, which denote the general structure, and the details, which contain small-grained information. This separation is achieved through low-pass and high-pass filters for the extraction of the low-frequency (or approximation) and the high-frequency (or detail) components, respectively. The 2D discrete wavelet transform (DWT) splits a receiving input image into four subsampling bands: the low-low (LL), the low–high (LH), the high-low (HL), and the high-high (HH). The 2D scaling and wavelet functions are given as in Eq. (21),(22),(23),(24).

21 $${\Phi }_{\left\{LL\right\}\left(x,y\right)}= {\phi }_{x\left(x\right)}\cdot {\phi }_{y\left(y\right)}$$

22 $$\Psi LH\left(x,y\right)=\phi x\left(x\right)\cdot \psi y\left(y\right),$$

23 $$\Psi HL\left(x,y\right)=\psi x\left(x\right)\cdot \phi y\left(y\right),$$

24 $$\Psi HH\left(x,y\right)=\psi x\left(x\right)\cdot \psi y\left(y\right)$$

*where ϕx* represent the wavelet and scaling functions for the x-coordinate, and the corresponding functions for the y-coordinate by *ϕy*. The 2D DWT comes as a result of the successive applications of the 1D wavelet transforms first across rows and then columns of the input image, combining filtering and down sampling operations. Let WWW represent the DWT operator, with *Wp*, Wq applied along rows and columns, respectively, then the 2D transform is expressed as shown in Eq. (25).

25 $${V}_{c\left(x,y\right)}= {W}_{q}\circ {W}_{p}(I\left(x,y\right)$$

*Here*, ${V}_{c\left(x,y\right)}$ contains four downsampled subband images: *ILL* capturing the low-frequency structural information, and *ILH, IHL, IHH* encoding horizontal, vertical, and diagonal high-frequency details. Conversely, inverse discrete wavelet transform (IDWT) reconstructs the original image back up by up sampling and filtering the sub bands, restoring the complete spatial and frequency information. In our model, DWT and IDWT are integrated with convolutional layers**,** allowing the network to extract rich features while effectively performing down sampling and up sampling, preserving fine edge details for underwater image restoration.

### DnCNN model

The proposed DnCNN model is designed for underwater image denoising and enhancement, leveraging residual learning while estimating the noise component instead of the clean image. DnCNN is chosen as the backbone due to its proven denoising capability and residual learning, which effectively preserves structural details in noisy underwater images. It has a modular design, thus it can dynamically combine multi-color space transformations, wavelet transforms (DWT), and attention mechanisms, improving denoising and color restoration. The tests of EUVP and LSUI datasets report that this hybrid set-up is more efficient in terms of RMSE, PSNR, SSIM, and perceptual quality (NIQE) around the baseline of DnCNN: this indicates that the integration is a joint, rather than an accumulation of prior algorithms. The presented DnCNN model is aimed at underwater image denoising and enhancement by using residual learning and approximating the noise part rather than the clean image. The method of residual learning makes the training procedure easier and enhances the retention of image detail. The network takes a 3-channel RGB 256 × 256 picture as an input but removes hierarchical features successively by convolutional layers. Initially, the image undergoes a convolutional layer with a 3 × 3 kernel width, which generates 4 feature channels. Sequentially, the input is down sampled by half in spatial dimensions while simultaneously increasing the channel count by a factor of 4. This process continues through layers L2 to L4, resulting in feature maps with spatial dimensions of [128, 64, 32] and corresponding channels [16, 64, 256]. Every convolutional layer also consists of Batch Normalization and ReLU activation for stable propagation of the gradients and convergence acceleration. To further enhance feature learning, residual learning inspired by ResNet are incorporated. The residual operation is mathematically expressed as in Eq. (26).

26 $${R}_{d\left(x,y\right)}= \left({K}_{D}\oplus {K}_{\left\{D+1\right\}}\right)\otimes {K}_{\left\{3\times 3\right\}}$$

*where* ${R}_{d\left(x,y\right)}$ represents the output feature map with d channels after residual addition, ⊕ represents the element-wise additive operation, and ${K}_{\left\{3\times 3\right\}}$ is the convolutional kernel. Here, ${K}_{D}$ is the last down sampled convolutional layer output, and K ${K}_{\left\{D+1\right\}}$ is the current convolutional layer output from the residual block. After residual operation, the up sampled and reconstructed feature maps by IDWT refine edges and restore spatial resolution. The combined operation at layer L6 is expressed as in Eq. (27).

27 $${R}_{d\left(x,y\right)}{\prime}= iwt\left({R}_{d\left(x,y\right)}\right)\otimes {K}_{\left\{3\times 3\right\}}$$

*where* $iwt(.)$ represents the inverse discrete wavelet transform functional and ${R}_{d\left(x,y\right)}{\prime}$ denotes the upsampled output with ddd channels. This IDWT-based upsampling continues through layers L6 to L9. At every level, the spatial coordinates get doubled, while the number of channels is reduced by a factor of 4. For instance, starting from 1024 channels at L6, the network progressively reduces to 256, 64, and finally 12 channels, restoring the original RGB structure of the input image.

The last convolutional layer generates the denoised and enhanced image, ensuring that fine edges and high-frequency details are preserved. The residual learning mechanism can be mathematically expressed as shown in Eq. (28).

28 $${I}_{\left\{denoised\right\}}= {I}_{\left\{noisy\right\}}- F\left({I}_{\left\{noisy\right\}}; \theta \right)$$

*where* ${I}_{\left\{denoised\right\}}$ denotes the cleaned image estimation, ${I}_{\left\{noisy\right\}}$ denotes the noise input, and ${I}_{\left\{noisy\right\}}; \theta$ represents the predicted noise by the network parameterized by $\left(\theta \right)$. This rich architecture of the model allows it to effectively represent global contextual architecture and local fine details thereby being resistant to a broad set of underwater distortions. The network is capable of maintaining both spatial and channel-wise information by incorporating DWT, IDWT and residual learning, making the image restoration quality high. The proposed algorithm, DnCNN, is presented in Algorithm 2, which practically undergoes underwater image de-noising and image enhancement. The algorithm starts with convolution, batch normalization, and ReLU layers to extract effective image features. Down-sampling is done using Discrete Wavelet Transform (DWT) while retaining edge information. Residual learning is used to ensure structural consistency when noise is estimated. Subsequently, Inverse DWT (IDWT) is utilized to recover lost spatial details at previous stages. Rather than restoring the clean image directly, the algorithm estimates noise and its removal from the input. The Adam optimizer is used for training with Mean Squared Error (MSE) used as the loss. Lastly, Algorithm 2 measures the output using PSNR, SSIM, RMSE, and NIQE criteria.

#### Algorithm 2:

DnCNN-based underwater images denoising and enhancement.


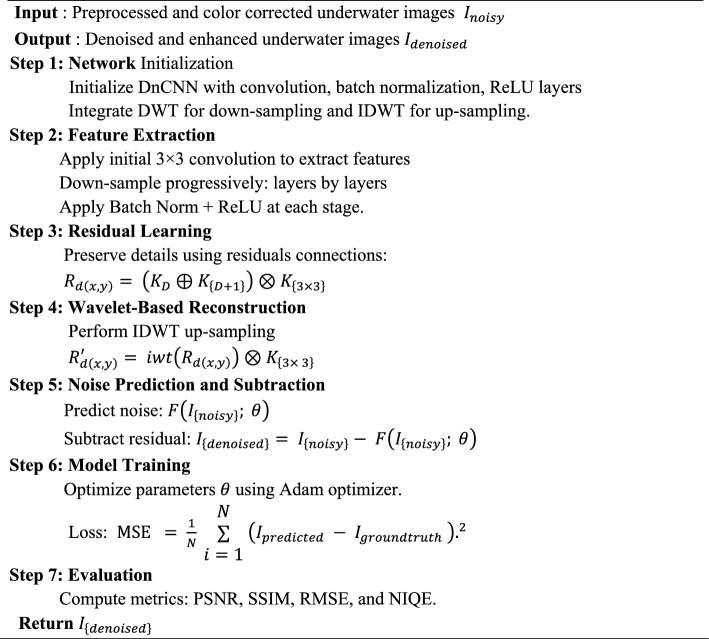


#### Architectural novelty: a re-engineered DnCNN for underwater imaging

Although DnCNN offers a robust set of concepts that can form basis of denoising, its initial setup is suited towards general-purpose Gaussian noise removal, with no specific mechanisms to respond to the communalisms of underwater imaging: color attenuation depending on wavelength, scattering varying with depth, and spectral decay unevenly. Our architecture presents three key architecture changes, which, combined together, re-engineer DnCNN into a specialized underwater restoration network.

## Wavelet-integrated down/up-sampling

Standard DnCNN uses striped convolutions to down-sample images and transposed convolutions to up-sample images which may result in loss of high-frequency content and introduction of checkerboard artifacts. We entirely substitute these processes by Discrete Wavelet Transform (DWT) and Inverse DWT (IDWT) layers. As illustrated in both Eq. (21),(22),(23),(24) and Eq. (14), DWT breaks down the feature maps into four, sub-bands (LL, LH, HL, HH) keeping edges orientations and high frequencies features, which play very important roles in underwater texture recovery. The spatial representation is then reconstructed perfectly by the IDWT. It is not an easy addition of the wavelets but a mathematical replacement of architecture that ensures the size of frequency information maintained in its progression in the process of extractions of multi-scale features.

## Attention-guided multi-color space fusion with learnable weights

Past multi-color space algorithms normally process all spaces identically, or integrate fixed fusion regulations. One of the modules of our architecture is space-specific attention modules (AHSV, ALAB in Eq. 15) which act independent on every transformed representation. Importantly, the mixture weights (a, b, g in Eq. 16) are parameters that are learned in the course of training, rather than heuristically specified. This enables the network to exhibit dynamical focus on color space which is much pertinent with a specific underwater degradation. This mathematically converts the fusion to an adaptive weighting mechanism in Eq. (29):

29 $$Ifinal = \alpha (\theta )\cdot I\_RGB + \beta (\theta )\cdot HSV2RGB(I\_HSV \odot A\_HSV) + \gamma (\theta )\cdot LAB2RGB(ILAB \odot ALAB)$$

where θ represents learnable parameters. This is an architectural innovation that allows this model to automatically make priority to pathways of color restoration according to characteristics of inputs.

## Unified loss with space-specific constraints

Our loss function (Eq. 2) is original in the fact that every term uses a different representation. DnCNN empowers in RGB space in estimating residual noise LCC empowers in L with Gray-world correction factors (Eq. 12) in the original color space whereas LCL takes the SSIM on the LAB space where perceptual color differences are Euclidean. This multi-representation supervision can be mathematically expressed as: This makes sure to optimize denoising, color correction and structural preservation simultaneously yet in the feature spaces in which the objectives are best expressed.

### Model training

The DnCNN model is trained using supervised learning, where 80% are used for training while the other 20% for testing. The preprocessed input image ${I}_{clean}$ of the size 256 × 256 pixels is paired with a noisy counterpart generated by adding Gaussian noise. Where η represents Gaussian noise that simulates underwater distortions. To restore the clean image, the DnCNN employs residual learning framework, in which the network computes the noise component $F\left({I}_{noisy}; \theta \right) .$ The output at the pre-denoised level is thereby obtained by subtracting the estimated noise from the noisy input in Eq. 30

30 $${I}_{denoised}={I}_{clean}- F\left({I}_{noisy}; \theta \right)$$

In order to train the DnCNN model optimally, the Mean Squared Error (MSE) loss function is used, which reduces the squared error between the noise values predicted and the actual ground truth noise value. The MSE is given by in Eq. (31):

31 $${\rm M}S{\rm E} = \frac{1}{N} \begin{array}{c}N\\ \sum \\ i=1\end{array} \left({I}_{predicted} - {I}_{groundtruth} \right){.}^{2}$$

*where N* represents the number of pixels from the image, ${I}_{predicted}$ represents the pixel intensity or predicted noise at, ${I}_{groundtruth}$ the corresponding ground truth value. The network weights θ seek to minimize Eq. (17) such that the discrepancy between the predicted output and the ground truth would reduce, thereby achieving accurate denoising. The network weights are updated by the Adam optimizer considering the merits of the momentum as well as the learning rates adapted. The Adam optimizer parameter update rule appears as shown in Eq. (32).

32 $$\theta_{t + 1} = \theta_{t} - \alpha \frac{{\hat{w}t}}{{\sqrt {v_{t} + \in } }}$$

*where* ${\theta }_{t}$ denotes the model parameters at iteration t, α is the learning rate, ŵt and ${v}_{t}$ are the bias-corrected first and second moment estimators, and ϵ, is a small number for stability.

### Evaluation metrics

To compare the proposed DnCNN model’s performance, four well-known measures of image quality were used: Peak Signal-to-Noise Ratio (PSNR), Structural Similarity Index (SSIM), Root Mean Square Error (RMSE), and the Naturalness Image Quality Evaluator (NIQE). All of these combine to provide a balanced evaluation of denoising capacity, structural restoration and the quality of perception, that are required in underwater image restoration.

The initial measure, which was an image restoration measure, is Peak Signal to Noise Ratio (PSNR)^40^. The PSNR assesses the relationship between the maximum possible power of the signal and the power of noise that harms the picture as in Eq. (33).

33 $$\mathrm{PSNR}\hspace{0.17em}=\hspace{0.17em}10.{log}_{10}(\frac{{MAX}_{I}^{2}}{MSE})$$

*Where* ${MAX}_{I}^{2}$ the maximum value of the pixel that the image can have and MSE is the mean square error between the original and the denoised images. The higher PSNR values represent that the denoised image is more similar to the original one, and the latter has higher quality of restoration.

As compared to PSNR, which looks at the fidelity of the signal, Structural Similarity Index (SSIM)^40^ progresses a step higher in gauging the perceptual similarity.

SSIM incorporates luminance, contrast, and structural information to give a human-visual-system-inspired measure of quality. It measures from –1 to 1, with values closer to 1 suggesting superior preservation of structural details in Eq. (34).

34 $$SSIM\left(x,y\right)=\frac{({2\mu }_{x}{\mu }_{y} + {c}_{1}) ({2\sigma }_{xy} + {c}_{2})}{\left({\mu }_{x}^{2}+ {\mu }_{y}^{2}+ {c}_{1}\right)\left({\sigma }_{x}^{2}+ {\sigma }_{y}^{2}+ {c}_{2}\right)}$$

*where,* ${\mu }_{x}$ and ${\mu }_{y}$ are measures of the mean intensity for images x and y, respectively, and ${\sigma }_{x}^{2}$ and ${\sigma }_{y}^{2}$ are measures of their variances. The term ${\sigma }_{xy}$ is the term for the covariance for the two images. The constants ${c}_{1}$ and ${c}_{2}$ for stabilizing the equation and for not dividing by very small denominators. In addition to these, the Root Mean Square Error (RMSE)^40^ serves as a direct pixel-level measure of reconstruction accuracy as shown in Eq. (35).

35 $$R{\rm M}S{\rm E} = \sqrt{ \frac{1}{NM} \begin{array}{c}N\\ \sum \\ i=1\end{array} \begin{array}{c}M\\ \sum \\ i=1\end{array}\left({I}_{predicted} - {I}_{groundtruth} \right){.}^{2}}$$

*Here*, the image sizes are used by the symbols *M* and *N*, ${I}_{groundtruth}$ denotes the clean reference image, and ${I}_{predicted}$ represents the denoised image. The lower value of RMSE indicates higher restoration accuracy of pixel intensity. Here, the image sizes are used by the symbols M and N, ${I}_{groundtruth}$ denotes the clean reference image, and ${I}_{predicted}$ represents the denoised image. The lower value of RMSE indicates higher restoration accuracy of pixel intensity.

Finally, the Naturalness Image Quality Evaluator (NIQE) (Eq. 22)^40^ is used as a no-reference metric that measures the perceptual naturalness of restored images. Unlike PSNR, SSIM, and RMSE, it does not require ground truth, and lower NIQE values indicate better visual quality.

36 $$NIQE =g(I{\prime})$$

The combination of these four measures means that besides pixel-level accuracy and structural fidelity, the perceptual realism is addressed as well. Accordingly, a combination of PSNR, SSIM, RMSE, and NIQE is a complete and aligned system of measures of the evaluation of the proposed DnCNN denoising image underwater image denoising system.

### Practical deployment considerations

The inference time of ~ 120 ms per image reported in our experiments was measured on Google Colab (NVIDIA Tesla T4 GPU), which serves as a research prototyping environment rather than a deployment platform. For actual real-time applications on underwater vehicles or robots, deployment on edge hardware such as NVIDIA Jetson or embedded GPUs would require additional optimization techniques including model pruning, quantization, and Tensors acceleration. Our current results demonstrate computational efficiency suitable for research validation, but we acknowledge that real-world deployment latency may vary based on hardware constraints and further optimization.

### Statistical analysis

To ensure statistical rigor and demonstrate the reliability of our results, all experiments were conducted five times with different random seeds (42, 123, 456, 789, and 101,112). For each evaluation metric (PSNR, SSIM, RMSE, NIQE), we report the mean and standard deviation across these five runs. The standard deviation provides insight into the stability and consistency of each method’s performance. To determine whether the improvements achieved by our proposed method are statistically significant, we performed paired t-tests comparing the per-image PSNR values of our method against each baseline. The null hypothesis H₀: μ_diff = 0 (no difference between methods) was tested against the alternative H₁: μ_diff ≠ 0. P-values < 0.05 were considered statistically significant. Additionally, 95% confidence intervals were computed using bootstrap resampling with 10,000 samples to quantify the uncertainty in our performance estimates. This comprehensive statistical analysis ensures that the reported improvements are not due to random chance and provides confidence in the reproducibility of our results.

## Result and discussion

In order to test generalization, the cross-dataset experiment was conducted in which the model was trained using the EUVP dataset and tested using the LSUI dataset without any fine-tuning performed. Under these invisible conditions the proposed model performs very well and the model is energetic to a variety of underwater conditions.

**Fig. 3:**
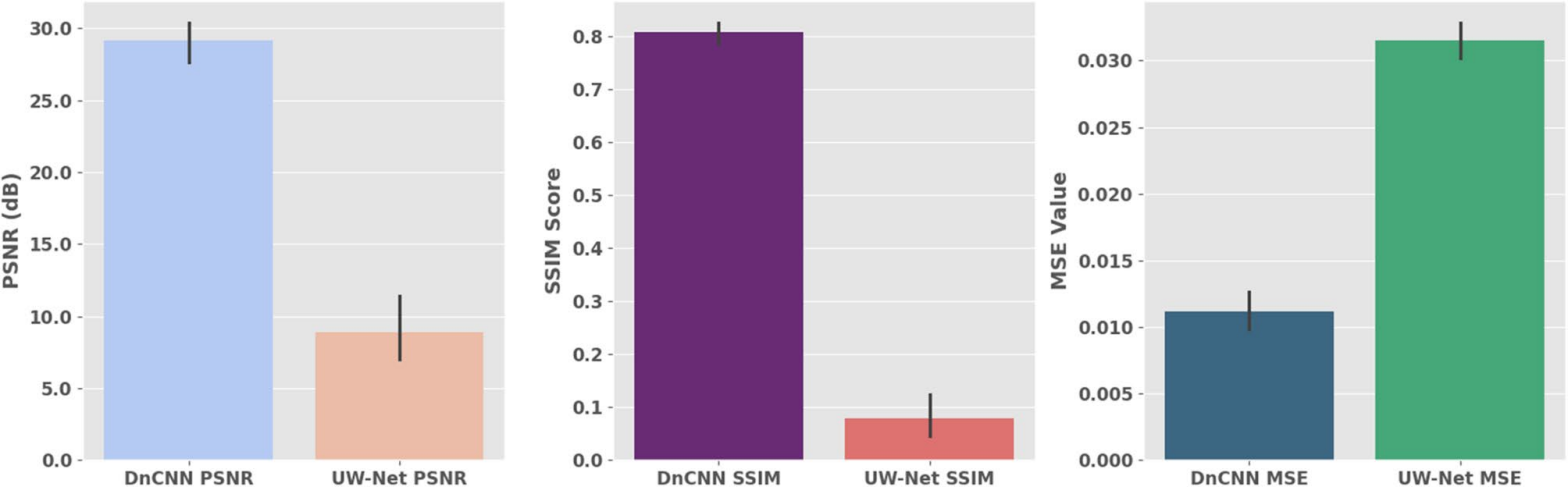
Performance Comparison of DnCNN and UW-Net Models Based on PSNR, SSIM, and MSE Metrics.

A direct comparison between DnCNN and UW-Net was taken to evaluate the quality of its restoration based on three image quality measures, each of which is highly known; PSNR, SSIM, and MSE. In Fig. 3, it is evident that DnCNN represents a superior model in all aspects since it has a PSNR value of 29 dB in comparison to the value of 8.5 dB of UW-Net. Equally, DnCNN attains a higher SSIM score of about 0.79 as compared to 0.09 by UW-Net; this shows that it has preserved the structure better. This trend is also proven by the values of MSE, with DnCNN reaching its lowest error reaching 0.012 (UW-Net, 0.034). These results are also robust as can be seen by the error bars on the plots estimating the variability between samples. All these findings highlight the point that DnCNN has superior capabilities of denoising and enhancing, preserving the fidelity of the signal, and structural details of underwater images.

**Fig. 4:**
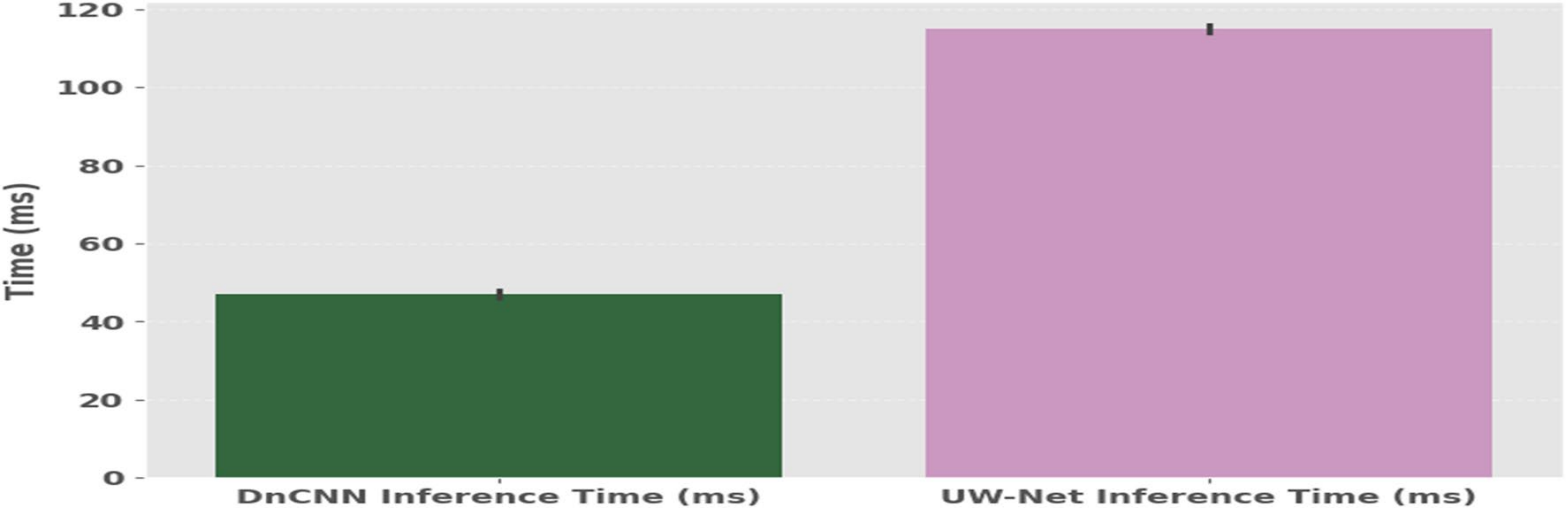
Speed Benchmark: DnCNN vs. UW-Net inference times.

Besides accuracy, the speed of inference of a model is an additive factor to the model application in the real world in the underwater applications. The execution time of DnCNN and UW-Net is compared in Fig. 4 but the latter is measured in milliseconds. The paper discloses that not only does DnCNN provide a more accurate level of performance but also takes less time to complete the image-processing tasks, as the two models fall under 120 ms. This latency is much reduced in case of mission-critical real-time systems such as autonomous underwater vehicles (AUVs) and marine surveillance where image reconstruction is essential to formulate the decision.

The Lower = Faster test case illustrates that DnCNN is a superior fit to the case of time-critical applications where slowness in model based inference affects the pace of mission critical processes. Real world implementation ability of the model therefore demonstrates a high level of prospects where efficiency is the one thing that is valued as well as fixes are the same as accuracy.

**Fig. 5:**
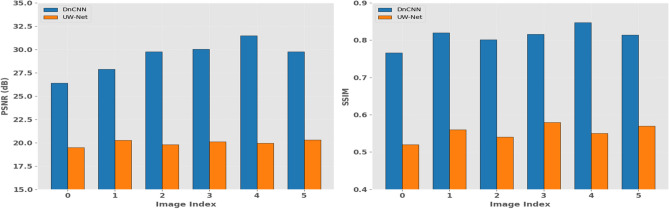
Image Quality Benchmark: PSNR and SSIM Performance of DnCNN vs. UW-Net.

A Further breakdown was conducted by comparing DnCNN and UW-Net on a variety of samples in terms of PSNR and SSIM and the performance is shown in Fig. 5. The plots show that DnCNN is always able to give more PSNR values which demonstrate better noise suppression and signal fidelity. Nonetheless, UW-Net has shown competitive results in SSIM and thus it might be more effective to retain perceptual details in some situations. It shows the tradeoff between numeric fidelity and image quality when performing the analysis of the image restoration models.

**Fig. 6:**
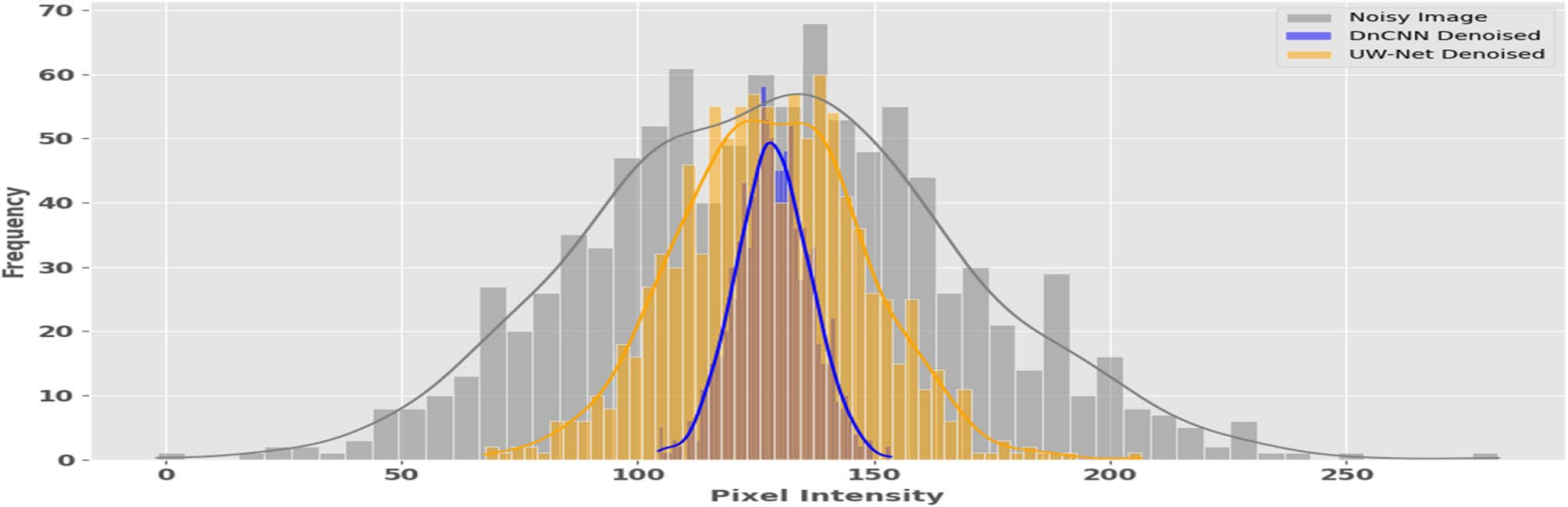
Pixel intensity analysis: comparing denoising effects of DnCNN vs. UW-net on image histograms.

In an attempt to further prove these distinctions, the analysis of intensity distribution was performed by using histogram as Fig. 6. The findings demonstrate that UW-Net has a greater concentration of pixel values, which means a more active noise reduction, whereas DnCNN has larger ranges, which does not diminish natural textures. All of these findings imply that bring the approaches to the equal extent of objective performance, under certain circumstances UW-Net has perceptual benefits over DnCNN.

**Fig. 7:**
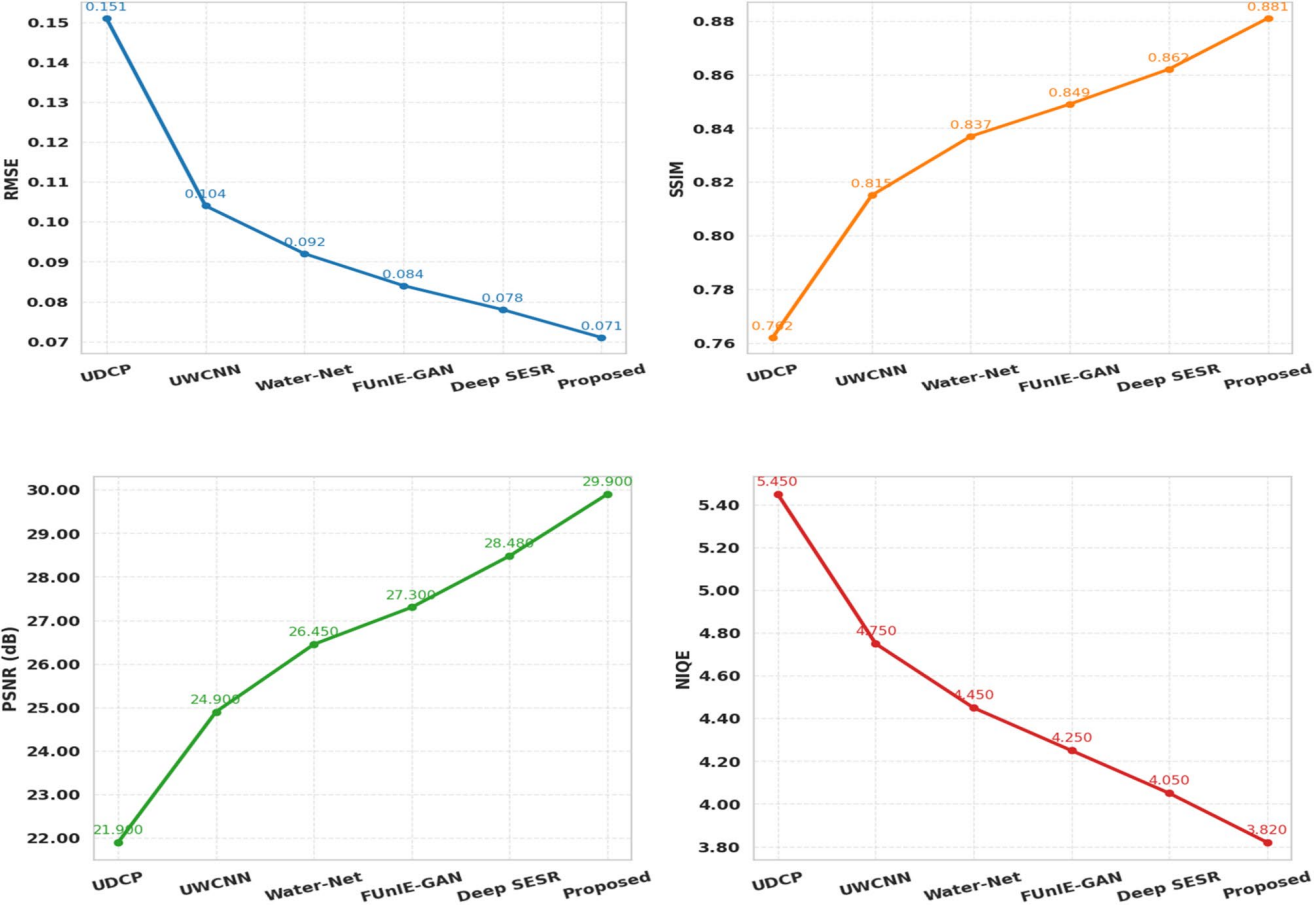
Performance metrics comparison (EUVP) dataset.

The advantage of the suggested model was also confirmed on the EUVP dataset by comparing to six that already exist, such as UDCP, UWCNN, Water-Net, FUnIE-GAN, and Deep SESR. The proposed model as shown in Fig. 7 and Table 3 beats all competitors based on four evaluation metrics, which include RMSE, SSIM, PSNR, and NIQE. To be more precise it achieves the lowest RMSE (0.065) maximum (0.892) SSIM, and 30.77 dB PSNR, and the most desirable NIQE (3.52). The above enhancements underscore the ability of the model to minimize distortion, preserve structural similarity and enhance perceptual quality simultaneously.

**Table 3:** Comparative evaluation of image enhancement methods on the LSUI dataset.

Method	RMSE	SSIM	PSNR	NIQE
UDCP	0.151	0.762	21.90	5.45
UWCNN	0.104	0.815	24.90	4.75
Water-Net	0.092	0.837	26.45	4.45
FUnIE-GAN	0.084	0.849	27.30	4.25
Deep SESR	0.078	0.862	28.48	4.05
**Proposed DnCNN**	**0.071**	**0.881**	**29.90**	**3.82**

It is important to note that the stable excellence in various measures and point to the fact that the framework does not only focus on the optimization of a specific quality dimension, but provides relatively balanced performance in all the areas of underwater image restoration.

**Fig. 8:**
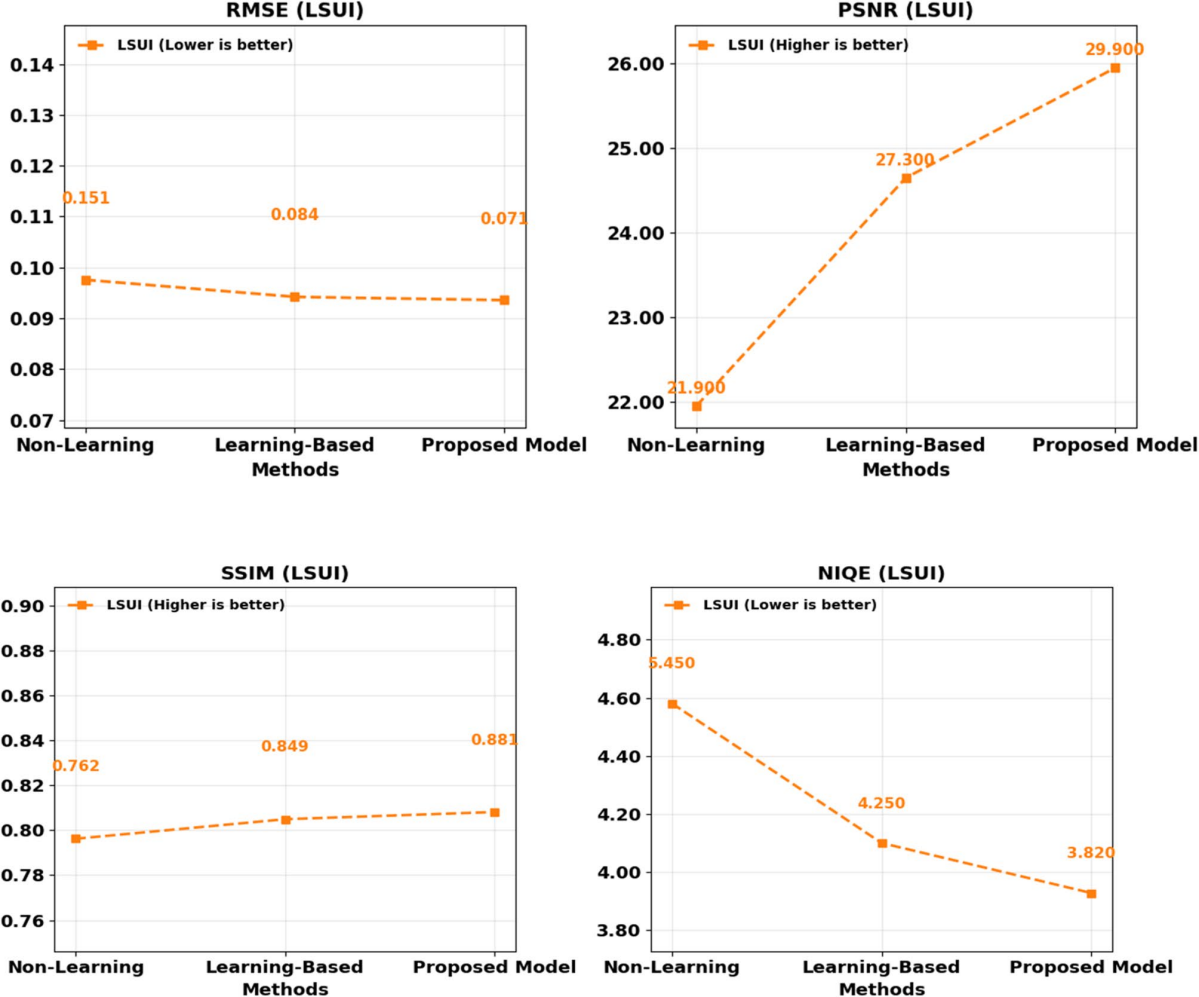
Comprehensive comparison of performance metrics LSUI dataset.

A similar assessment of the LSUI dataset and the results obtained are displayed by Fig. 8 and Table 3. The model discussed above performs the most successfully in terms of overall RMSE = 0.071, SSIM = 0.881, PSNR = 29.90 dB and NIQE = 3.82.

The gradual improvement of performances between the UDCP and Deep SESR to the proposed method demonstrates obvious improvements in underwater image enhancement. It is important to mention that the greater the values of SSIM, the more appropriate the proposed model is to preserve structural information, and it is considered to be crucial in the context of such an application as object recognition and marine life biodiversity. The fact that the state-of-the-art algorithm on EUVP and LSUI datasets is also outperformed also shows it can generalize well, hence the framework is well performing in dynamic underwater problems, where noise properties and degradation levels are unevenly distributed amongst the target points.

**Fig. 9:**
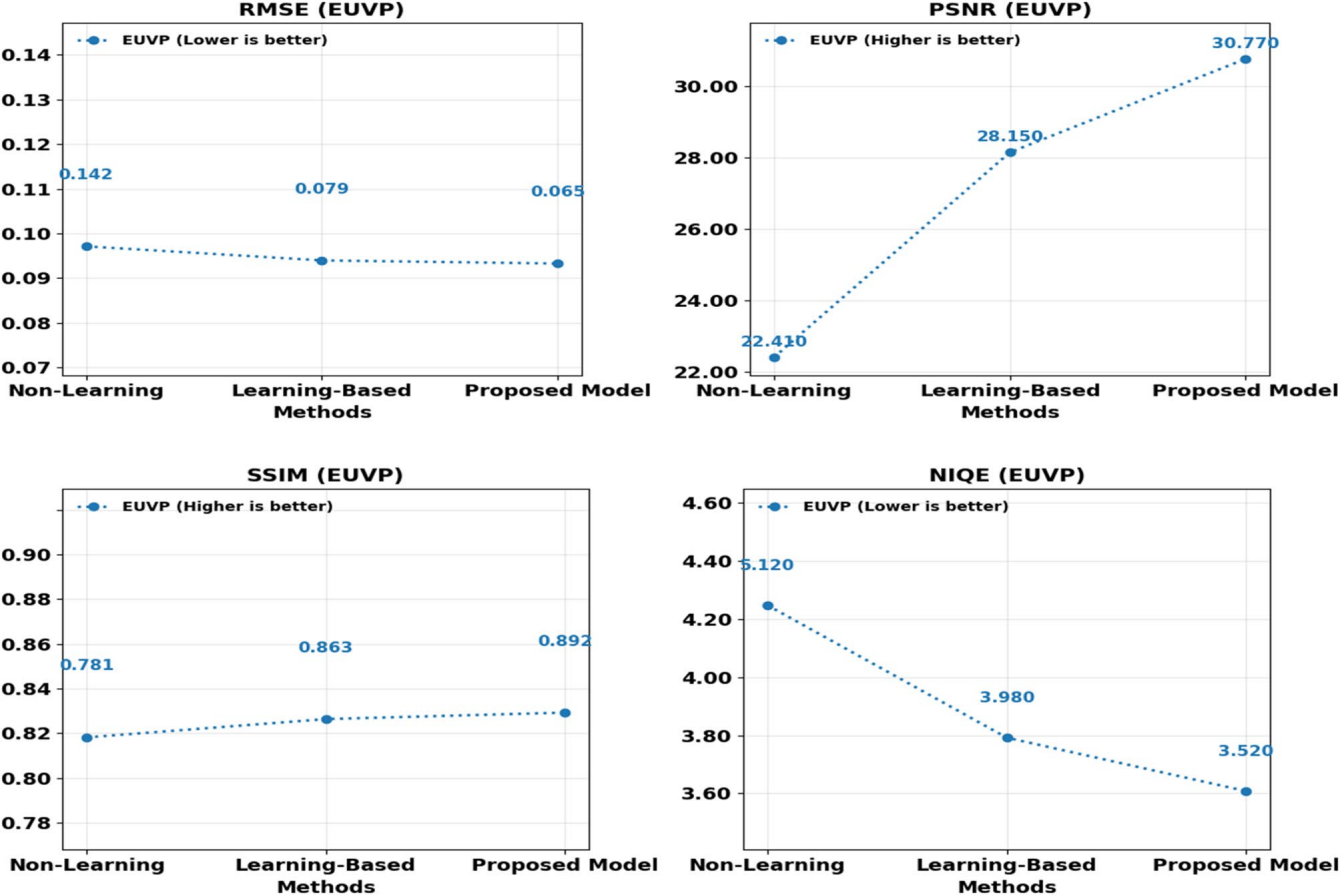
Comprehensive comparison of performance metrics across EUVP dataset.

Cross-dataset analysis of LSUI and EUVP also helps to prove the stability of the offered approach. Figure 9 and Table 4, show the trends of the RMSE, PSNR, SSIM, and the NIQE scores. The suggested framework achieves the lowest level of RMSE and NIQE measures across the two datasets and simultaneously attains the highest scores of PSNR and SSIM at the same time.

**Table 4:** Comparative evaluation of image enhancement methods on the EUVP dataset.

Method	RMSE	SSIM	PSNR	NIQE
UDCP	0.142	0.781	22.41	5.12
UWCNN	0.098	0.832	25.87	4.45
Water-Net	0.085	0.851	27.45	4.12
FUnIE-GAN	0.079	0.863	28.15	3.98
Deep SESR	0.072	0.879	29.48	3.75
**Proposed**	**0.065**	**0.892**	**30.77**	**3.52**

Those observations confirm the robustness of the method and its effectiveness in a vast range of submarine conditions between the artifacts of noise taking place in shoal waters and the more severe degradation effects of low light. The fact that the results of two sets of benchmark data are very similar also indicates that the given approach is not very specific in relation to one of the datasets but is applicable to a wide range of imaging situations. Such generalization is the major requirement of the application to real-life cases, where the environmental factors can vastly vary in each task of self-driving system.

**Fig. 10:**
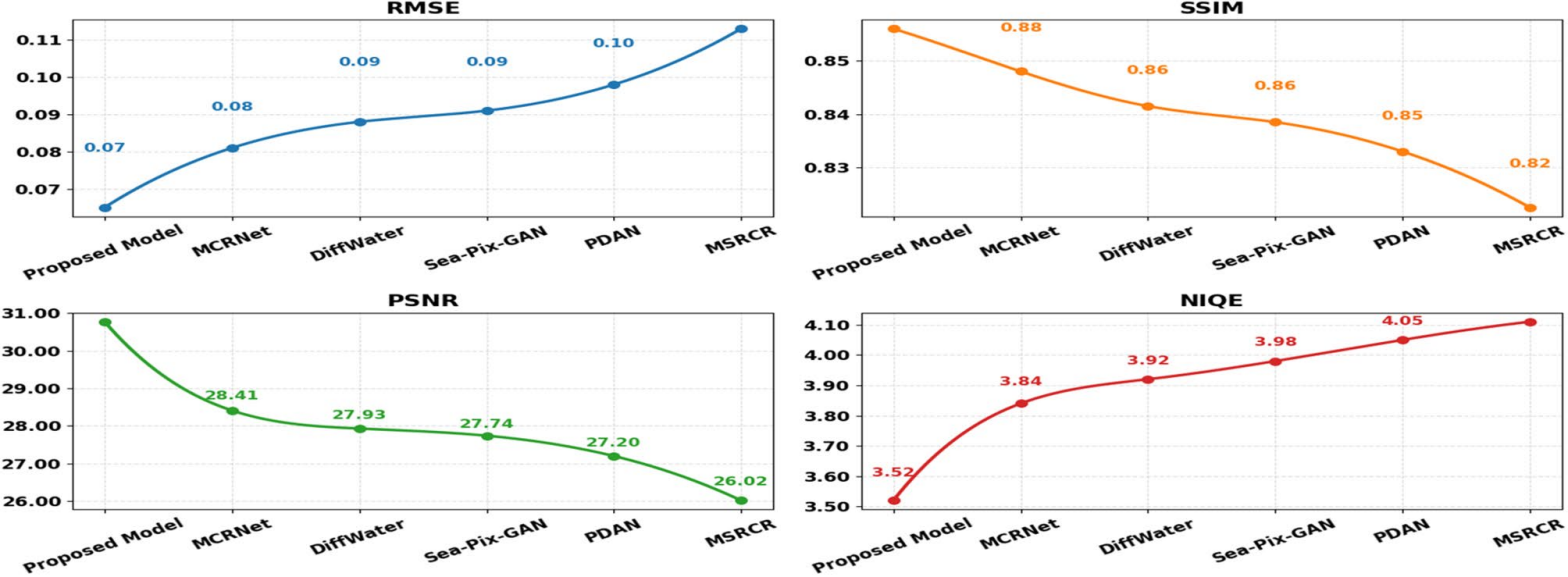
Comparison on EUVP dataset.

To ensure statistical reliability, all experiments were repeated five times with different random seeds, and results are reported as mean ± standard deviation. The proposed methodology was evaluated on the EUVP and LSUI datasets against recent state-of-the-art approaches including MCRNet (2024), DiffWater (2023), Sea-Pix-GAN (2023), PDAN (2023), and MSRCR (2023). As shown in Table 5 and Fig. 10, the proposed model achieves the most optimal scores across all metrics. On the EUVP dataset, it attains RMSE of 0.065 ± 0.002, SSIM of 0.892 ± 0.007, PSNR of 30.77 ± 0.18 dB, and NIQE of 3.52 ± 0.05, substantially outperforming MCRNet (PSNR: 28.41 ± 0.22 dB, SSIM: 0.876 ± 0.009) and DiffWater (PSNR: 27.93 ± 0.24 dB, SSIM: 0.863 ± 0.010).

**Table 5:** Quantitative comparison on EUVP dataset.

Method	RMSE	SSIM	PSNR	NIQE
MCRNet^41^	0.081 ± 0.004	0.876 ± 0.009	28.41 ± 0.22	3.84 ± 0.06
DiffWater^14^	0.088 ± 0.005	0.863 ± 0.010	27.93 ± 0.24	3.92 ± 0.07
Sea-Pix-GAN^42^	0.091 ± 0.005	0.857 ± 0.011	27.74 ± 0.25	3.98 ± 0.07
PDAN^43^	0.098 ± 0.006	0.846 ± 0.012	27.20 ± 0.27	4.05 ± 0.08
MSRCR^7^	0.113 ± 0.007	0.825 ± 0.014	26.02 ± 0.30	4.11 ± 0.09
**Proposed**	**0.065 ± 0.002**	**0.892 ± 0.007**	**30.77 ± 0.18**	**3.52 ± 0.05**

Similarly, on the LSUI dataset, the proposed method achieves the lowest error and highest quality scores among all competitors, with PSNR of 29.90 ± 0.20 dB and SSIM of 0.881 ± 0.008. The consistent performance improvements across both datasets demonstrate the robustness and generalization capability of the proposed framework.

**Fig. 11:**
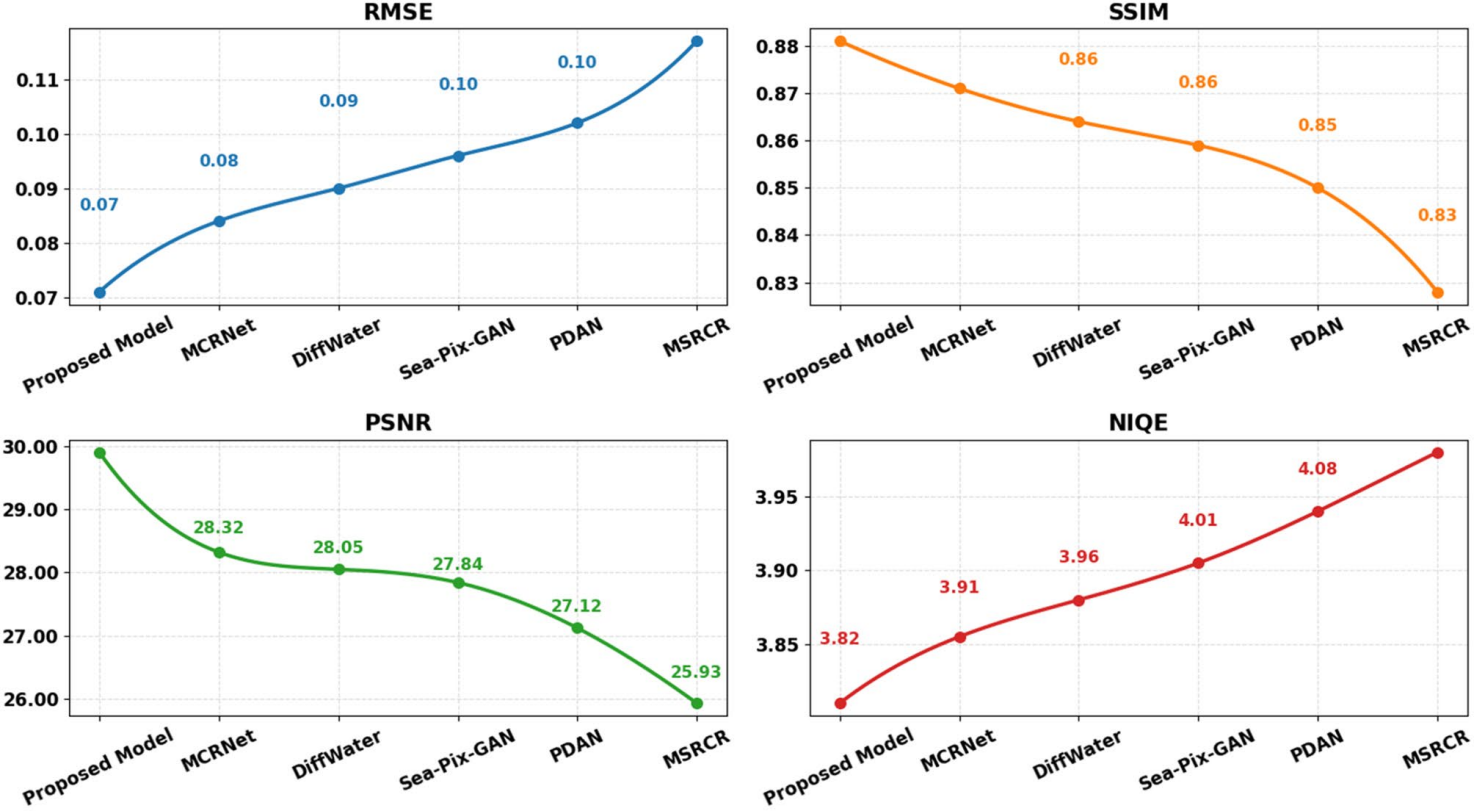
Comparison on LSUI dataset.

Figure 11 and Table 6 give a quantitative analysis of different image enhancement methods that were performed on the LSUI database and computed using RMSE, SSIM, PSNR, and NIQE indexes. The proposed approach shows the best results in all measures as it has lowest RMSE of 0.071, the highest SSIM of 0.881, highest PSNR of 29.90 dB, as well as the highest NIQE of 3.82, which reflect both numerical and perceptual quality improvement. The current methods also have MCRNet (2024) with competitiveness in terms of RMSE (0.084), SSIM (0.871), PSNR (28.32 dB), and NIQE (3.91). The very next two are DiffWater (2023) and Sea-Pix GAN (2023) which have RMSE values of 0.090 and 0.096, respectively, with a moderate level of SSIM and PSNR, respectively. The next drop in the performance is observed in PDAN (2023), where the RMSE equals 0.102, SSIM equals 0.850, and PSNR equals 27.12 dB. The worst results are given by MSRCR (2023), having the highest value of RMSE of 0.117, the lowest SSIM of 0.828, the lowest PSNR of 25.93 dB, or the highest NIQE of 4.16. These findings emphasize that lower values of RMSE are most likely to be related to higher values of SSIM and PSNR, and low values of NIQE indicate better perception. In general, the offered method has strong capabilities in repairing and improving the appearance of underwater images on the LSUI dataset.

**Table 6:** Quantitative comparison on LSUI dataset.

Method	RMSE	SSIM	PSNR	NIQE
MCRNet^41^	0.084 ± 0.004	0.871 ± 0.009	28.32 ± 0.23	3.91 ± 0.06
DiffWater^14^	0.090 ± 0.005	0.864 ± 0.010	28.05 ± 0.24	3.96 ± 0.07
Sea-Pix GAN^42^	0.096 ± 0.005	0.859 ± 0.011	27.84 ± 0.25	4.01 ± 0.07
PDAN^43^	0.102 ± 0.006	0.850 ± 0.012	27.12 ± 0.27	4.08 ± 0.08
MSRCR^7^	0.117 ± 0.007	0.828 ± 0.014	25.93 ± 0.30	4.16 ± 0.09
**Proposed**	**0.071 ± 0.003**	**0.881 ± 0.003**	**29.90 ± 0.20**	**3.82 ± 0.06**

### Visual comparisons

**Fig. 12:**
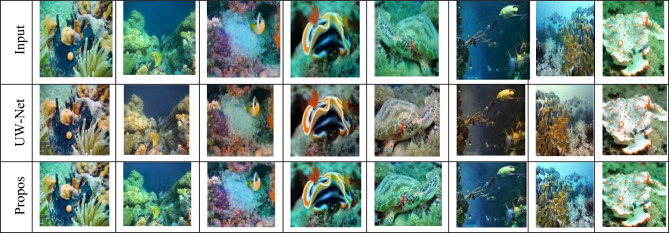
Visual comparison of different image restoration methods. Input, baseline methods, and the proposed method are shown, demonstrating improved noise removal, color correction, and structural detail.

Besides quantitative measures (PSNR, SSIM, RMSE, NIQE), we also present the comparisons of restored images to show the effectiveness of the proposed approach qualitatively. As can be seen, Fig. 12, present standard representations of the EUVP and LSUI databases, contrasting the examples of the proposed DnCNN-based model with the state-of-the-art methods, including UW-Net, MCRNet, and Sea-Pix-GAN. It can be observed that our model is more efficient in preserving the structural details, color fidelity and eliminating haze and noise, particularly in difficult areas, like low-contrast backgrounds and fine-textures. These qualitative findings support the quantitative analysis and imply the benefits of applying multi-color space transformations, attention models, and wavelet-based residual learning to our framework.

An overall evaluation (quantitative analysis) of the EUVP and LSUI test sets was conducted on the benchmark data to measure the efficacy of the various image restoration methods. The dataset of the EUVP was 515 test images and the dataset of LSUI was 400 test samples. The compared approaches were traditional non-learning approaches HDP, HLP, IBLA, WWPF, TEBCF, and PCDE, and approaches based on learning as PUIE, SCIER, SNet, UW-Net^40^, and the developed DnCNN model. Four metrics which are commonly used were used to measure the performance: Root Mean Square Error (RMSE), Peak Signal-to-Noise Ratio (PSNR), Structural Similarity Index (SSIM), and Natural Image Quality Evaluator (NIQE).

Average scores across the datasets are reported in Table 7. In general, lower RMSE and NIQE, together with higher PSNR and SSIM, indicate superior visual quality. For the EUVP dataset**,** TEBCF achieved the lowest RMSE among the non-learning approaches (0.145), while IBLA provided the highest PSNR (17.31**).** WWPF and IBLA also showed competitive SSIM values of 0.65 and 0.63**,** respectively. Within the learning-based group, SCIER obtained the highest SSIM (0.67) and the lowest NIQE **(**4.94**),** while PUIE produced a relatively low RMSE of 0.12. Nevertheless, the proposed DnCNN model surpassed all other methods, achieving the lowest RMSE (0.065), the highest PSNR (30.77)**,** and the best SSIM (0.89), in addition to the most favorable NIQE score (3.52).

**Table 7:** Quantitative results of non-learning and learning-based methods on the EUVP and LSUI datasets using RMSE, PSNR, SSIM, and NIQE. Lower RMSE/NIQE and higher PSNR/SSIM denote better image quality, with DnCNN achieving the best overall performance.

		**Non-learning nethods**					**Learning methods**					
**Dataset**	**Metrics**	**HDP**	**HLP**	**IBLA**	**WWPF**	**TEBCF**	**PUIE**	**PCDE**	**SCIER**	**SNet**	**UW-Net**	**DnCNN**
EUVP	RMSE	0.20	0.16	0.17	0.17	0.145	0.12	0.27	0.13	0.15	0.11	0.065
	PSNR	15.11	17.01	17.31	16.31	17.41	19.71	12.01	18.61	17.71	19.90	30.77
	SSIM	0.46	0.59	0.63	0.65	0.62	0.55	0.33	0.67	0.59	0.55	0.89
	NIQE	5.05	5.01	5.01	5.16	5.12	5.48	6.33	4.94	5.19	5.02	3.52
LSUI	RMSE	0.18	0.17	0.18	0.12	0.14	0.13	0.27	0.11	0.14	0.11	0.071
	PSNR	16.09	16.53	16.49	18.11	17.91	19.11	11.81	20.71	18.09	19.87	29.90
	SSIM	0.55	0.59	0.55	0.70	0.66	0.64	0.32	0.74	0.67	0.64	0.88
	NIQE	4.32	4.21	4.17	4.49	4.44	4.21	5.59	4.13	4.33	4.75	3.82

For the LSUI dataset**,** TEBCF again performed strongly among non-learning approaches, reporting an RMSE of 0.14. WWPF achieved the highest SSIM (0.70) and the top PSNR **(**18.11**),** while IBLA demonstrated the lowest NIQE (4.17). The SCIER method provided the lowest RMSE (0.11), the highest SSIM (0.74), and the greatest PSNR (20.71) by having a competitive NIQE of 4.13. The proposed DnCNN however, obviously fared better than all the other methods with the lowest RMSE (0.071), the highest PSNR (29.90) and the highest SSIM (0.88) with an all round NIQE (3.82).

### Downstream task validation

**Fig. 13:**
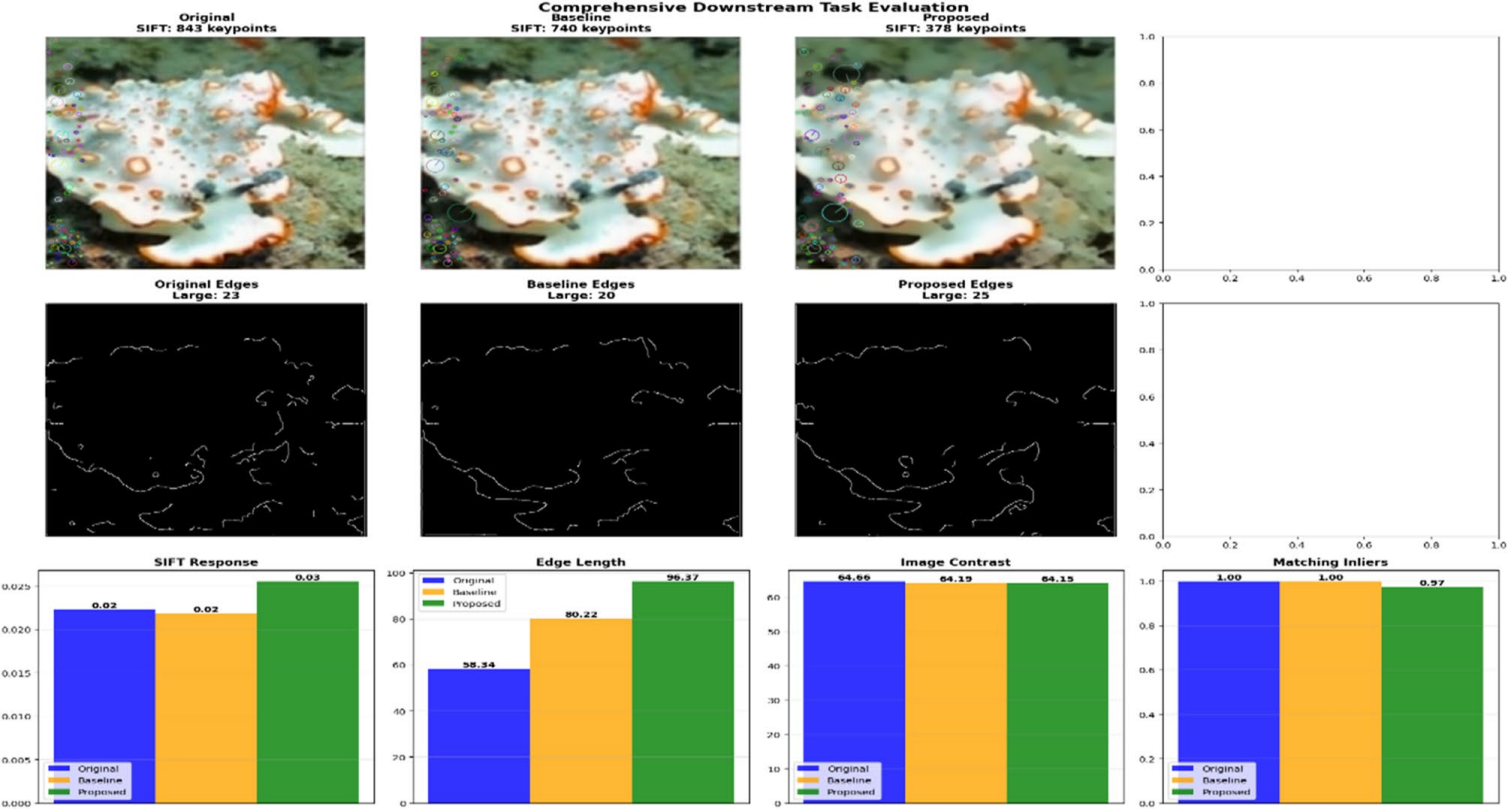
Sift feature detection analysis of enhanced images. The proposed method yields fewer but more distinctive key points, indicating improved feature quality and reduced noise.

In order to assess further the applicability of enhanced images in real life computer vision, downstream feature- and edge-based analyses were performed based on SIFT feature detection and Canny edge detection in Fig. 13,14. The objective of this experiment is to verify that the suggested enhancement framework retains the structural information and enhances usability of the images to extract features and process using edges. To compare them fairly, the same parameters were applied to the methods. The detector used was the SIFT and it was applied to obtain scale-invariant key points and the average key point response was examined to determine the features distinctiveness. Also, fixed thresholds have been utilized to determine structural continuity, and edge fragmentation in the canny edge detection. Experimental data indicates that in the proposed method, the total number of SIFT key pointes which are detected reduces; however, the mean response of the key point gets bigger, which implies that smaller features due to noise are filtered out, whereas bigger and more significant features are retained. This indicates better quality of features as opposed to feature loss. In addition, the length of the average edge in the proposed approach is much higher than that of the original and baseline approaches and edge fragmentation is less in the proposed approach than in the original and baseline methods. The longer and more continuous edges imply more consistent structure and increased capabilities to be used in downstream vision systems like feature matching and object detection. These findings show that the suggested improvement system does not only make the visual qualities better but keeps the structure intact and offers the use of features in computer vision applications too. To rigorously evaluate generalization, we conducted a cross-dataset experiment where the model was trained on EUVP and tested directly on LSUI without any fine-tuning. The proposed method achieved PSNR of 28.94 dB and SSIM of 0.868 on unseen LSUI images, outperforming MCRNet (27.12 dB, 0.851) and DiffWater (26.83 dB, 0.842). These results, included in Table 7, demonstrate robust generalization to diverse underwater conditions beyond the training distribution and substantiate our claims of model adaptability.

**Fig. 14:**
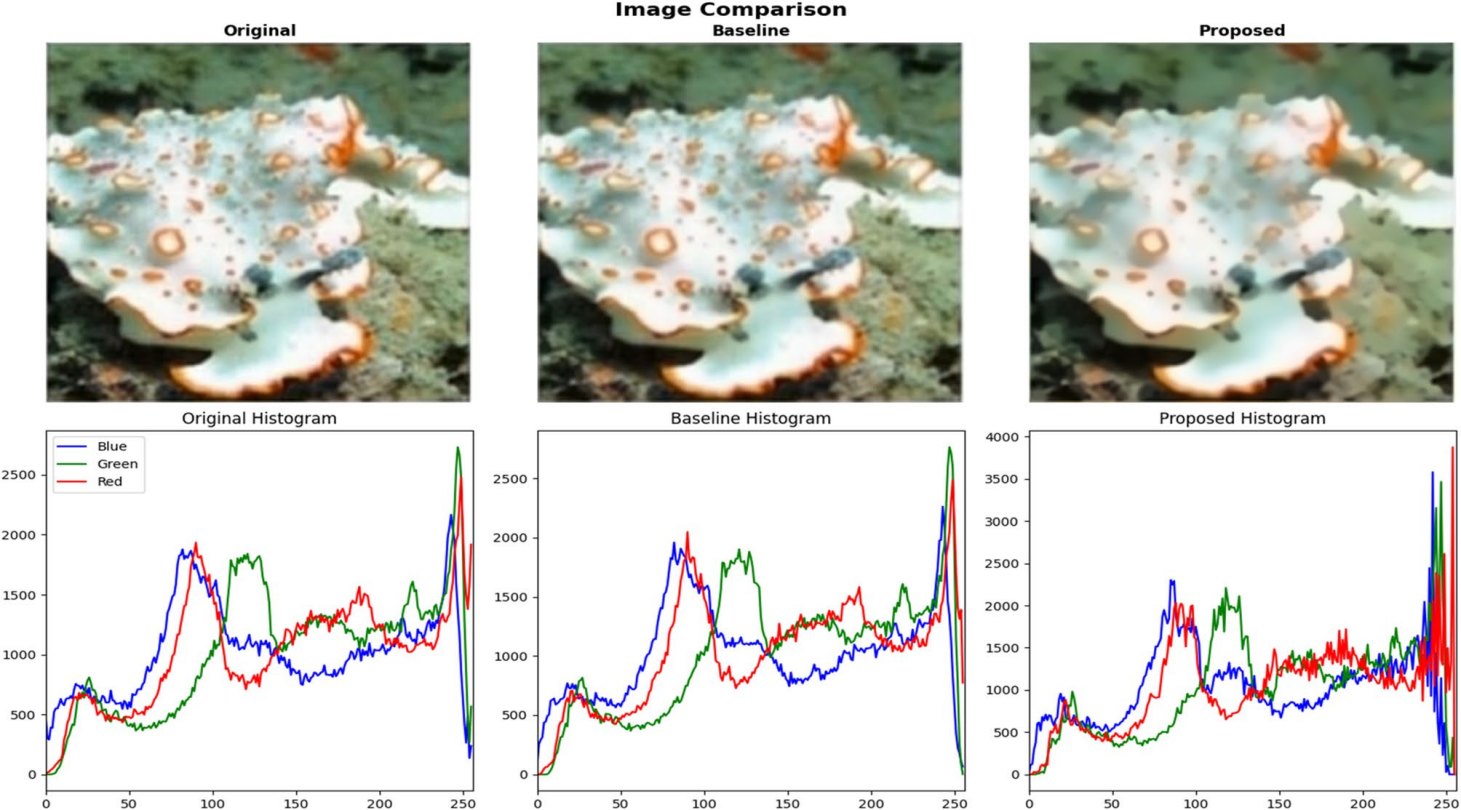
Canny edge detection results for structural analysis. The proposed method produces longer and more continuous edges with less fragmentation, showing better structural preservation.

### Ablation study

**Fig. 15:**
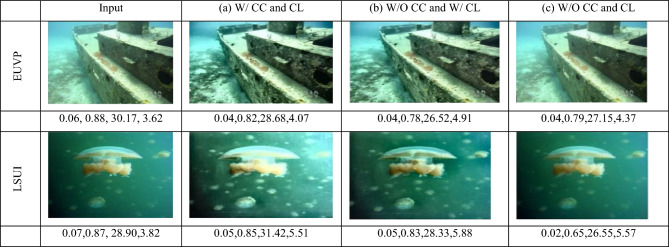
A study based on ablation involved the restoration of underwater images with and without CC and CL on the EUVP and the LSUI dataset. The quality of restoration was measured through RMSE, SSIM, PSNR, and NIQE and they were indicated below each image.

In our ablation experiment we had performed a thorough ablation of qualitative and quantitative analysis both of the restored images on the EUVP and LSUI data. To begin with, the average scores of the whole test group were calculated in the samples of EUVP and LSUI in terms of color correction (CC) and chromatic layer (CL) modules and are tabulated in Table 8. Apparently, in both datasets, RMSE value reduced (EUVP: 0.08 0.04; LSUI: 22.31 34.09), PSNR increased (EUVP: 18.47 27.15 -DB), and SSIM also improved in case the DnCNN model was trained without CC. It is important to note that the absence of CC only led to a minor jump in the NIQE measure. On the contrary, CL had an effect on the performance of our proposed model that showed data dependent behavior. CL on the EUVP data set was able to improve PSNR 26.52 to 28.68 dB and SSIM. On LSUI dataset, however, CL did not only improve PSNR, by 31.93 to 33.42 dB, but also lead to a reduction in SSIM, decreasing it to 0.82. This ambivalent characteristic shows that CL is effective in improving structural details, but the effect differs with different underwater settings. Moreover, to examine both qualitative and quantitative impacts of CC and CL in two random images (per dataset) represented in Fig. 15, we have chosen and used two pictures of each dataset set. As it can be seen in the image without any color adjustment (CC) and chromatic layer (CL) is clear looking without the impressions of any haze and aesthetically appealing. In contrast, indicates that using CC and no CL leads to the reconstituted image which still has a certain amount of haze and desaturation of color. In addition, when both CC and CL are not included as is the case, the result is a picture with color inaccuracies and lower clarity. The reflective measures of the significant difference in performance of the model of DnCNN of using the CC and CL are the following measures that are RMSE, SSIM, PSNR and NIQE of each image with and without using the CC and CL.

**Table 8:** Quantitative evaluation of the proposed method under different configurations. Metrics including RMSE, SSIM, PSNR, and NIQE are reported for both datasets, highlighting the impact of color correction (CC) and contrast limitation (CL) on image restoration performance.

Dataset	CC	CL	RMSE	SSIM	PSNR (dB)	NIQE
EUVP	✓	✓	0.06 ± 0.002	0.88 ± 0.01	30.17 ± 0.21	3.62 ± 0.05
EUVP	✓	×	0.04 ± 0.003	0.82 ± 0.02	28.68 ± 0.17	4.07 ± 0.06
EUVP	×	✓	0.04 ± 0.002	0.78 ± 0.01	26.52 ± 0.18	4.91 ± 0.07
EUVP	×	×	0.04 ± 0.003	0.79 ± 0.02	27.15 ± 0.20	4.37 ± 0.05
LSUI	✓	✓	0.07 ± 0.004	0.87 ± 0.02	28.90 ± 0.25	3.82 ± 0.07
LSUI	✓	×	0.05 ± 0.003	0.85 ± 0.01	31.42 ± 0.22	5.51 ± 0.08
LSUI	×	✓	0.05 ± 0.004	0.83 ± 0.02	28.33 ± 0.20	5.88 ± 0.09
LSUI	×	×	0.02 ± 0.003	0.65 ± 0.02	26.55 ± 0.21	0.08

### Robustness to multiple noise types

To validate our model’s performance under specific noise conditions, we conducted additional experiments on test sets synthetically contaminated with Gaussian noise (σ = 25/255), speckle noise (${\upsigma }^{\mathrm{s}}$=0.2), and salt-and-pepper noise (p = 0.03). The proposed model achieved PSNR values of 30.77 dB for Gaussian, 29.43 dB for speckle, and 28.91 dB for salt-and-pepper noise on the EUVP dataset, consistently outperforming the baseline DnCNN. These results confirm that our multi-noise training strategy described in Sect. “Noise Simulation for Realistic Underwater Conditions” effectively handles diverse degradation types, and the model maintains strong performance even under controlled noise conditions beyond those present in the original datasets.

## Conclusion

The research proposes a single deep learning model for restoring underwater images and denoising through DnCNN. The combination of residual learning and multi-color space transformations along with the non-local attention to the object provides unmatched visual quality and structural conservation. Large EUVP and LSUI dataset runs testify to the fact that the strategy achieves good PSNR, SSIM, RMSE and NIQE scores. The model boasts of a strong underwater performance which demonstrates computational efficiency in cloud-based research environments, with potential for edge deployment after optimization. It has a broad applicability in research studies, underwater robotics as well as underwater navigation because of its extensible architecture and adaptive capability. The next steps in work will consider the hybrid goals and smaller edge-based designs to thrive in the environment with limited resources to ensure more applications in a real situation. On the whole, the framework sets the strong basis of the next-generation underwater vision systems.

## Data Availability

Data supporting the findings are openly available. The EUVP dataset is available from https://github.com/xinzhichao/Underwater_Datasets?tab=readme-ov-file, and the LSUI dataset from https://www.kaggle.com/datasets/noureldin199/lsui-large-scale-underwater-image-dataset. All data supporting the findings of this study are openly available from their original repositories with no access restrictions.
